# Exploring craniofacial fluctuating asymmetry in a South African sample

**DOI:** 10.1111/joa.14256

**Published:** 2025-04-23

**Authors:** Miksha Harripershad, Leandi Liebenberg, Alison F. Ridel, Charlotte E. G. Theye

**Affiliations:** ^1^ Department of Anatomy University of Pretoria Pretoria South Africa; ^2^ Laboratoire de Paléontologie, Évolution, Paléoécosystèmes et Paléoprimatologie (PALEVOPRIM) UMR 7262 CNRS & Université de Poitiers Poitiers France

**Keywords:** fluctuating asymmetry, geometric morphometric methods, micro‐focus X‐ray computed tomography, South African population

## Abstract

Biological anthropologists have extensively investigated the relationship between fluctuating asymmetry (FA) and its association with population history and health. However, in South Africa, few studies have been done on skeletal asymmetry and its potential impact on anthropological analyses. The study aimed to examine craniofacial asymmetry within a contemporary South African population, specifically focusing on the association between FA, sex, and population affinity. The sample consisted of cranial micro‐focus X‐ray computed tomography scans (micro‐XCT) from 115 adult individuals. Craniometric landmarks were placed and used to calculate inter‐landmark distances (ILDs) to assess size variation, and geometric morphometric methods (GMMs) were performed to assess asymmetrical shape variation. Additionally, two indices assessing FA (FA8 and FA17) were calculated from the left and right ILDs to further explore patterns of asymmetry for various regions of the cranium. Asymmetry was particularly apparent in females and black South Africans, which is consistent with reports in the literature. Significant levels of FA were noted in the nasal and temporal regions, more so for shape than size. While asymmetry potentially may have a minimal influence on biological profile estimations based on craniometry, biological anthropologists should have an understanding of the consequences asymmetry may have on skeletal elements or traits being employed on an individual basis.

## Graphical abstract

This study investigated craniofacial fluctuating asymmetry (FA) in a contemporary South African sample using micro‐XCT scans. Inter‐landmark distances (ILDs) assessed size variation, while geometric morphometric methods (GMMs) captured shape variation. FA was higher in females and black South Africans, particularly in the nasal and temporal regions, emphasising the need to consider FA in anthropological analyses.

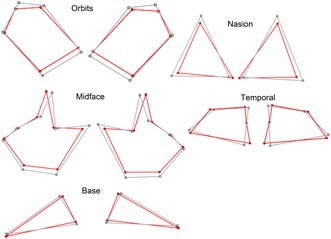

## 1 INTRODUCTION

Skeletal asymmetry refers to inequality in the size and/or shape of bilateral (left and right sided) anatomical structures and is often studied by biological anthropologists for a variety of applications, such as exploring developmental stress, effects of skeletal asymmetry on the biological profile, etc. (Cole et al., 2020; Graham & Özener, 2016; Yaussy, 2024). The literature defines asymmetry into three types: directional asymmetry, anti‐symmetry, and fluctuating asymmetry (FA) (Graham & Özener, 2016; Van Valen, 1962; Willmore et al., 2007). Directional asymmetry is a constant growth or enlargement of a bilateral trait to one side, to such an extent that it is common amongst the majority of the population, with larger dimensions typically on the dominant side. The mean distribution between the right and left sides is not equal to zero due to the unequal right and left side development (Graham & Özener, 2016). Directional asymmetry is influenced by behavioral actions such as handedness (dominant limb usage), which is linked to the locomotive behavior during childhood (Willmore et al., 2007). As such, right‐hand dominance is common in the majority of the population because of the constant use of one side over the other, resulting in asymmetry. Right bias is frequently observed in the postcranial skeleton of the general population (right side traits being larger than left side traits) (Auerbach & Ruff, 2006; Hagg et al., 2017; Latimer & Lowrance, 1965). Anti‐symmetry is defined as bilateral traits with one side being more developed. However, unlike directional asymmetry, anti‐symmetry is random and not caused by pattern limb dominance (Palmer, 1994; Van Valen, 1962). Thus, the distribution of anti‐symmetric bilateral trait development within a population will be variable; half of the population will exhibit right side dominance while the other half will exhibit left side dominance (Palmer, 1994; Willmore et al., 2007). Graham and Özener (2016) discuss anti‐symmetry using lobster claws as an example, in which 50% of the individuals had larger right claws and 50% had larger left claws, while only a few had equal sized ones. The distribution of the mean of anti‐symmetry is bimodal and the mean is close to or equal to zero (Graham & Özener, 2016). Finally, FA is defined as a random deviation from perfect symmetry with an inequality in the size or shape of bilateral traits and has a symmetrical distribution of values around a mean of zero (Graham & Özener, 2016; Palmer, 1994; Van Valen, 1962). It arises primarily due to the effects of developmental stress and instability, stemming from genetic and environmental stressors (Graham & Özener, 2016; Özener, 2022; Zakharov & Trofimov, 2022). FA can also occur spontaneously as a result of random developmental processes (Graham & Özener, 2016; Klingenberg, 2015; Özener, 2022). Additionally, bioanthropological and bioarcheological studies have examined the potential of FA as an indicator of early‐life stress, stipulating that early‐life stress can have a major impact on an individual's health and development (Moes et al., 2022; O'Donnell & Moes, 2021; Yaussy, 2024). FA often occurs as the result of energy being diverted for short‐term survival rather than for growth and development during periods of stress (Moes et al., 2022; O'Donnell & Moes, 2021; Yaussy, 2024). Therefore, FA can be used to gauge developmental instability linked to adult health outcomes observable in human skeletal remains at a population level.

Various skeletal elements are influenced by FA, exhibiting varying degrees of FA. However, certain bones, such as those in the upper and lower limbs, may exhibit directional asymmetry which could also inflate FA values. Compared with postcranial elements, the cranium lacks a substantial pattern of one‐sided dominance (Graham & Özener, 2016; Hagg et al., 2017; Harripershad et al., 2024), is thus less affected by directional asymmetry, and can assist in providing a better gauge of FA in individuals (DeLeon, 2007; Graham & Özener, 2016; Weisensee & Spradley, 2018). Bilateral traits or anatomical landmarks, such as the dacryon (in the orbits), the horizontal plates of the palatine bones, or pterions (intersection between frontal, temporal, parietal, and sphenoid bones), are all suggested to be more sensitive to stress and therefore show a greater degree of craniofacial FA (Hagg et al., 2017). Storm and Knüsel (2005) examined various cranial traits from two Medieval English populations to determine which traits had the greatest magnitude of FA. The results showed that the mastoid breadth and height, orbital breadth, and bregma‐porion height were the most asymmetrical traits, while the orbital height and zygomatic height showed the least FA. Hallgrímsson (1999) proposed that cranial regions that fuse later in development, such as the face, will show higher levels of FA due to decreased developmental stability. By contrast, Gawlikowska et al. (2007) as well as DeLeon (2007) observed that in modern populations, FA was mostly observed in the cranial vault and base, with low levels of FA noted in the facial region. Hagg et al. (2017) also reported high levels of FA in the cranial base. Among different populations, patterns of craniofacial FA may thus vary, and more research is still needed to understand how the magnitude of FA is patterned across the cranium in the South African population.

### 1.1 FA and the South African context

Developmental instabilities are described as an individual's inefficiency to produce a desired phenotype (expected phenotype for a given genotype and environment) under a specific environmental condition, such as nutritional deficiencies, adverse living conditions, extreme temperatures, or genetic mutations (Nijhout, 2003; Palmer, 1994). The term “stressor,” often used in biological anthropology, refers to an event resulting in physiological change caused by strain placed upon an individual brought about by environmental and genetic conditions (Reitsema & McIlvaine, 2014). Often in childhood, stress may arise from congenital defects, living in poverty, and/or poor health due to susceptibility to disease. Research has shown that children from low socioeconomic backgrounds tend to be hospitalised more frequently, have an increased susceptibility to disease throughout their lifetime, and demonstrate higher degrees of FA (Livshits et al., 1988; Palmer, 1994). Özener and Fink (2010) also noted that developmental instabilities related to poor living conditions often manifest as craniofacial FA. Notably, studies have explored how FA levels may vary between socioeconomic groups in human populations. These studies have highlighted the impact cultural changes, and social factors of environmental fluctuations can have on measures of developmental instability (Bigoni et al., 2013; DeLeon, 2007; Harripershad et al., 2024; Livshits et al., 1988; Özener & Fink, 2010; Weisensee & Spradley, 2018; Yaussy, 2024).

The diverse nature and history of the South African population yield an ideal sample to explore FA in the craniofacial complex, the impact of socioeconomic status (SES) on measures of developmental instability, and the potential impact on forensic applications. Historical issues within southern Africa have resulted in long‐lasting socioeconomic disparities between populations. Racial inequality was present long before apartheid, and black South Africans were already living under dire circumstances following the colonisation and slavery brought on during the establishment of the Cape Colony (Worden, 2023). Persistent inequalities and unemployment in the population largely stemmed from institutional racism associated with the apartheid regime (1948–1994), which prevented people of color from having access to good education and stable employment (Daniel, 2020; Stoddard, 2022). The apartheid regime created a wealth and power imbalance between racial groups, which continues to persist (Fogel, 2019; Linford, 2017). Presently in South Africa, an estimated four million South Africans are experiencing multidimensional poverty (e.g., poor health, malnutrition, and lack of access to basic needs), which is in part due to high levels of unemployment in the country (Daniel, 2020; Mariotti & Fourie, 2014; Stoddard, 2022). In 2021, 61% of people of color in South Africa were estimated to be living in poverty, while only 1% of the white South African population is experiencing poverty (Government Gazette, 2021). As a result, many black and colored South Africans still have inadequate access to health care and are highly susceptible to disease (Daniel, 2020; Stoddard, 2022).

This study focusing on FA in a South African sample will provide information on how SES‐related factors will affect the development of craniofacial FA while also addressing geographic and demographic gaps.

### 1.2 The influence of FA on biological parameters

An understanding of asymmetry is required in forensic anthropology as it may potentially influence estimates of the biological profile resulting in misclassification (Cole et al., 2020). Asymmetry has previously been shown to influence the biological profile, particularly when assessing stature and age estimates, as well as when identifying the number of individuals in comingled remains (Corron & Stull, 2017; Kanchan et al., 2008; Krishan et al., 2010; Nandi et al., 2018; Živanović, 1983). Furthermore, both cranial and pelvic fluctuating and directional asymmetry were reported to have implications on sex estimates with regard to the biological profile (Cole et al., 2020). As a result, anthropologists may benefit from a better understanding of the magnitude of asymmetry expressed by an individual, as it is imperative to gain accurate biological profile estimates (Cole et al., 2020). A good grasp of craniofacial FA in the South African population and among individuals can therefore benefit anthropologists. However, FA in the contemporary South African population has rarely been explored (Harripershad et al., 2024; Holland, 2015; Kieser & Groeneveld, 1988).

With regard to population affinity estimation (where population affinity is defined as an individual's resemblance to particular groups of interest that share similar morphological and genetic traits, Ross & Pilloud, 2021; Spradley & Jantz, 2021), research on asymmetrical population differences is greatly needed, not only to better comprehend the understanding of population history and instability but also to assist the processes of facial approximation and identification in the forensic field. Since different populations experience varying stressors, assessing the extent of FA in populations is crucial. However, only a small number of studies have been conducted with regard to population differences and the craniofacial regions affected by FA (Kieser & Groeneveld, 1988; Schlager, 2013). When examining FA on the hard tissue of the nose, Schlager (2013) found that, compared with the Chinese population, the European population had considerably more variation and a statistically significant higher degree of FA. Such information on FA was extremely useful and important to consider before the reconstruction of the soft tissue of the nose for facial approximation purposes, specifically in the European population studied (Schlager, 2013). Furthermore, due to the socioeconomic constraints experienced among South Africans by means of population stressors (e.g., apartheid and poverty), research by Kieser and Groeneveld (1988) found that black South Africans exhibited higher levels of dental FA as opposed to the white South African population. This great degree of FA in the South African population may potentially be attributed to the high prevalence of disease and malnutrition in this population group following the legacy of apartheid (Kieser & Groeneveld, 1988).

The evaluation of sexual dimorphism may be influenced by FA, as high levels of developmental instability may alter the differential development of sexually dimorphic traits, which should thus be considered when estimating the biological profile (Cole et al., 2020). Females are thought to be more resistant to developmental instabilities due to their genotype. More specifically, XX‐chromosomes can buffer deleterious recessive alleles, while the single X chromosome present in the male genotype (XY sex chromosomes) cannot (Graham & Özener, 2016). Consequently, females are considered more “stable,” and males are predicted to express a greater degree of asymmetry (Graham & Özener, 2016). Furthermore, research has shown that males and females have demonstrated differences in immune responses (Fish, 2008; Klein & Flanagan, 2016), that are in part related to hormones, social background, and X‐linked genes. Females tend to have a greater resilience to infection and developmental stressors due to the immune‐regulating effects of estrogen, as opposed to males who tend to be more susceptible to disease and developmental stressors (Fish, 2008). These factors can cause sex‐based variations resulting in differences in FA between sexes in different populations. Literature discusses a “maternal buffering system,” which is a physiological response to physical stress among females (Reck et al., 2016). The maternal buffering system plays an integral role in fetal nutrition and has been proposed to decrease the harmful effects of nutritional stress on the mother throughout pregnancy while increasing the benefits of dietary supplements taken during pregnancy (Kuzawa & Fried, 2017). When a female is pregnant, the buffering system will work to protect (or buffer) the infant, so that the infant is able to receive adequate resources for in utero growth and develop sufficiently, while the mother is able to absorb all environmental stressors that may otherwise be exerted on the infant. Should the mother herself experience stressful conditions while developing, her buffering system will not be as efficient. When the mother is pregnant and experiences stressful conditions, the fetus will be susceptible to environmental stressors (Howell et al., 2017; Reck et al., 2016), consequently increasing developmental instability and FA levels in the child. In this way, the generational effects of poverty and poor living conditions are observed (Howell et al., 2017) as the maternal buffering capacity would become inadequate and result in both the mother and fetus not being protected by environmental stressors effectively leading to high levels of FA in both the mother and fetus. In the literature, studies have been inconsistent in describing the relationship between sex and FA. In a recent living Turkish sample, Özener and Fink (2010) noted sex differences in facial FA, but only in males living in lower socioeconomic conditions. Similarly, Hope et al. (2013) found higher levels of craniofacial FA in males than in females as a result of SES in a sample consisting of 292 individuals from the Lothian Birth Cohort 1921 (LBC1921). Lastly, Teul et al. (2024) also examined FA in 877 Polish university students (483 women, 394 men) using six bilateral body traits and found that lower SES and higher air pollution levels during early development were linked to greater FA, with higher levels in men than in women. However, other studies on various prehistoric and contemporary populations found no significant sex differences for FA when evaluating the crania, mandible, dentition, and postcranial skeleton (Costa, 1986; Hallgrímsson, 1999; Perzigian, 1977; Townsend & Garcia‐Godoy, 1984). Owing to the myriads of contradictory results and inconsistencies in evaluating the relationship between sex and FA, more research is needed to understand the extent to which asymmetry may differ between the sexes, especially in a South African population.

### 1.3 Research goals and hypotheses

This study aimed to explore craniofacial asymmetry in a contemporary South African population using craniometric landmarks collected on micro‐XCT scans. The presence and magnitude of asymmetry within and between populations and sexes were then assessed. This study used a multivariate approach to analyse both the asymmetry of size and of shape as they are complementary components of morphological variation. Asymmetrical size variation was examined via cranial measurements (obtained from inter‐landmarks distances), while asymmetrical shape variation was assessed directly from the landmark coordinates. Therefore, the following hypotheses were tested:

1. Black South Africans are hypothesised to exhibit higher levels of FA than white South Africans due to historically greater population stressors (e.g., poverty, apartheid, SES).
2. South African males are hypothesised to express a higher degree of FA compared with their female counterparts due to their biology and exposure to more stressors in their cultural environment.
3. The facial area as well as the base of the cranium will show high levels of FA, according to the literature.

## 2 MATERIALS AND METHODS

### 2.1 Sample

The sample consisted of micro‐focus X‐ray computed tomography (micro‐XCT) scans of 115 crania (59 black and 56 white South Africans, comprising 57 females and 58 males) from individuals sourced from the Pretoria Bone Collection (PBC), housed in the Department of Anatomy, University of Pretoria, South Africa (Table 1). The PBC is a skeletal collection of South African individuals, with known records of birth dates (min‐max birth years: 1863–1996), age, sex, and population affinity (obtained from identification documents), that were unclaimed or donated to the University of Pretoria medical school (Krüger et al., 2015; L'Abbé et al., 2005, 2021; Liebenberg et al., 2019). The individuals included in the sample were born between 1882 and 1980, with death years ranging between 1964 and 2012. The sample thus includes individuals that grew up and were alive during colonisation of southern Africa, slavery, and the apartheid regime (1948–1994) (L'Abbé et al., 2005, 2021). Cranial scans for this research were obtained from the *Bakeng se Afrika* repository, housed at the University of Pretoria (L'Abbé et al., 2021). The micro‐XCT scans were performed at the South African Nuclear Energy Corporation (Necsa, Pelindaba, South Africa) using a Nikon XTH225L industrial Computed Tomography system (Nikon Metrology, Belgium). The parameters of scanning were as follows: 100 kV voltage; 100 μA current; 1000 projections per 360°; and isotropic voxel sizes ranging from 100 to 110 μm.

**TABLE 1:** Study sample distribution.

	Black South Africans	White South Africans	Total
Females	30	27	57
Males	29	29	58
Total	59	56	115

Only well‐preserved crania of adults (age range: 21–89; mean age: 53 ± 16 years) were selected, and any individuals with trauma, bone alterations, or metal restorations that could affect scanning or the ability to collect measurements accurately were excluded. There are many covariables that can influence the face, and more precisely asymmetry of the face, and these variables are intricately linked. In this particular study, we focused on sex and population, given the limitations of the sample. It should be acknowledged that age likely plays a significant role on the presence of FA. However, consideration of age as a variable requires thorough ontogenetic assessment from childhood to adulthood, and thus, it was beyond the scope of the study to explore age as a covariable. Furthermore, Palestis and Trivers (2016) and Chovalopoulou et al. (2017) found that adult age does not have an impact on craniofacial FA. In addition, the age distribution in the sample is balanced between the population groups, with white South Africans being slightly older (median = 62); as a result, age is not expected to have a huge bearing on the results of this study. Ethical approval to conduct this research was obtained by the University of Pretoria Faculty of Health Sciences Research Ethics Committee (Ethics Number: 386/2021).

### 2.2 Methods

Prior to any analysis, the micro‐XCT scans were segmented (i.e., virtual extraction of the region of interest) using the semi‐automatic watershed method available in the 3D imaging software Avizo 2019 (ThermoFisher Scientific Inc.). Segmentation allows the generation of 3D models that were used in the subsequent analyses. All 3D models were re‐oriented and aligned according to the standard Frankfort plane, which is a standard anatomical reference plane often used in anatomy and anthropology to orient the cranium (White & Folkens, 2005). The orientation was performed by placing four anatomical standard landmarks: the right and left porion (located at the most superior point along the upper margin of the external acoustic meatus), and the right and left orbitale (most inferior point on the lower orbital rim) (Caple & Stephan, 2016; Langley et al., 2016). Once all crania were in the standard Frankfort position, 34 craniometric landmarks were manually placed (see Harripershad et al. (2024) for more detailed descriptions and images). To evaluate both the asymmetry of size and of shape, asymmetry was assessed from the landmarks in two ways: (1) by calculating inter‐landmark distances (ILDs) and related FA indices, providing, respectively, information on general size‐based craniofacial asymmetry, or size‐based FA statistical information; (2) using geometric morphometric methods (GMMs), which allow for a subtle shape‐based assessment of craniofacial FA compared with physical measurements (Benítez et al., 2020; Klingenberg, 2015). This multivariate approach allows for a comprehensive assessment of asymmetrical variation and FA in the cranium and the South African population.

**FIGURE 1:**
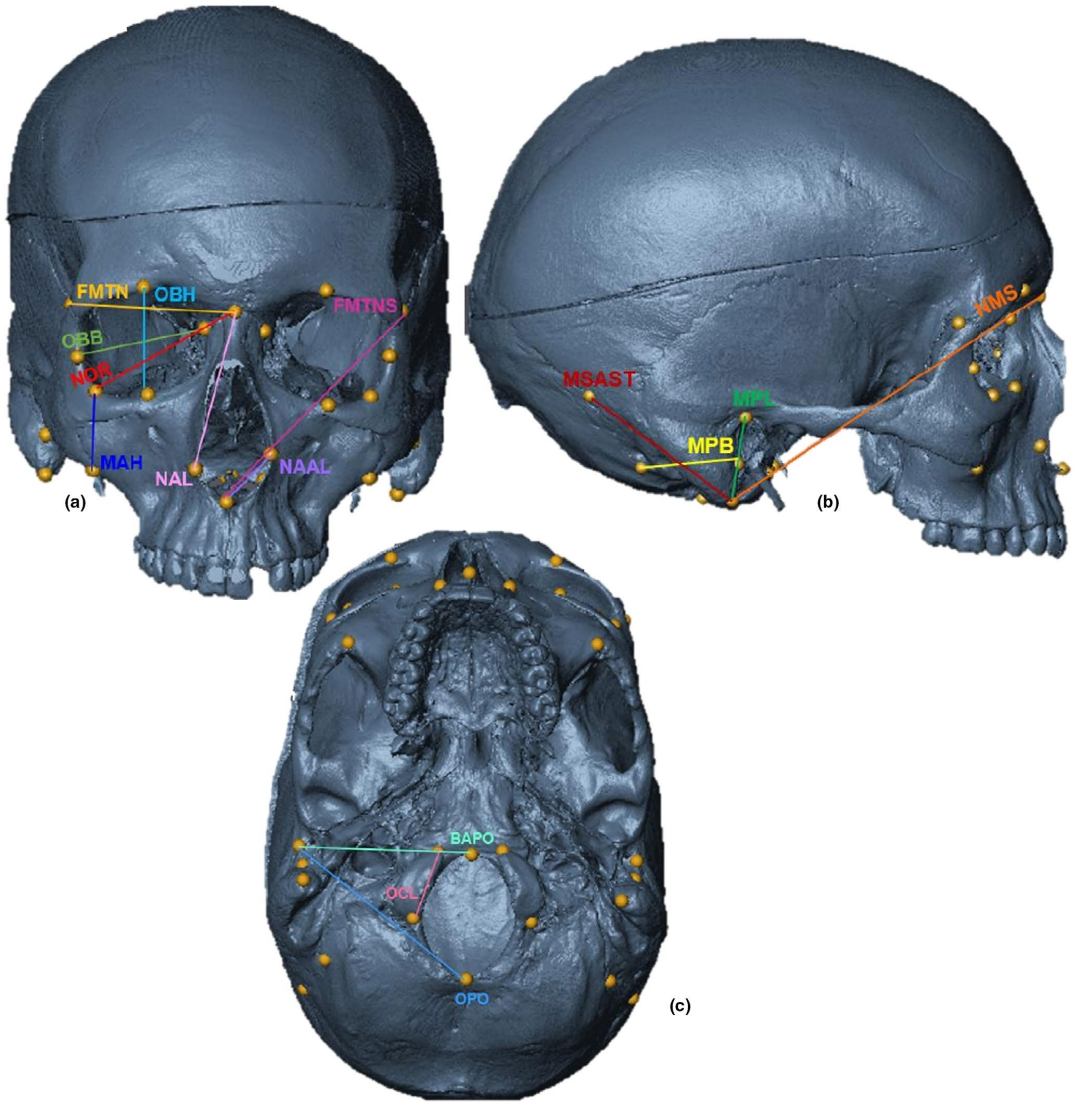
Landmarks (1–34) placed on the cranium with the inter‐landmark distances (ILD) taken (cf. Table 2 for definitions). (a) Frontal view: OBB (blue), OBH (purple), NOR (red), FMTN (yellow), FMTNS (dark pink), MAH (dark blue), NAL (pink), NAAL (purple); (b): lateral view: MPL (green), MPB (yellow), MSAST (red), NMS (orange); (c) inferior view: OCL (pink), OPO (blue), BAPO (green). BAPO, basion‐porion length; FMTN, frontomalare‐nasion length; FMTNS, frontomalare‐nasospinale; MAH, malar height; MPB, mastoid breadth; MPL, mastoid length; MSAST, mastoidale‐asterion; NAAL, nasospinale‐alare; NAL, nasion‐alare; NMS, nasion‐mastoidale; NOR, diagonal orbital breadth; OBB, orbital breadth; OBH, orbital height; OCL, occipital condyle length; OPO, opisthion‐porion length.

The 15 ILDs calculated (refer to Table 2 for definitions and Figure 1 for visualisation of the distances) are standardised measurements commonly employed in skeletal analyses following internationally recognised standards of best practice (Langley et al., 2016). FA indices (left side measurement minus right side measurement) were calculated from each ILD for each individual. Since fluctuating indices can increase with trait size, a standardised scale needed to be used to assess asymmetry, allowing for an accurate comparison between traits with different magnitudes. Standard asymmetry formulae (FA8 and FA17 indices) developed by Palmer and Strobeck (2002) were thus used. The FA8 index (Formula 1) is univariate and assesses asymmetry per ILD, while the FA17 index (Formula 2) is a multivariate combination of all FA8 indices. Different combinations of FA8 indices were used to calculate several FA17 indices and represent specific cranial regions (e.g., orbital, face; see Table 3 for the definitions). Essentially, FA8 provides asymmetry estimates on univariate cranial measurements, while FA17 provides an overall (multivariate) average of asymmetry pertaining to the entire cranium and for a combination of region‐specific cranial measurements. Thirty‐four landmarks were also used to assess shape variation among the different cranial regions to assess which cranial regions exhibited the most FA in the overall cranium and between population and sex groups. Different combinations of landmarks (reflecting the cranial regions of the FA17 indices) illustrating different configurations (i.e., matrices of the midface, orbital region, nasion, base and temporal regions) were constituted and analysed.

**TABLE 2:** Inter‐landmark distances (ILDs) calculated from the craniometric landmarks (adapted from Hagg et al., 2017; Harripershad et al., 2024). All ILDs were calculated for the left and right sides. See Figure 1 for landmarks and ILDs visualisation.

Name	Region	Abbreviation	Definition
Orbital breadth	Orbits, Midface	OBB	Distance from the dacryon (D) to ectoconchion (Ec)
Orbital height	Orbits, Midface	OBH	Distance between the superior (So) and inferior (Io) orbital margins
Diagonal orbital breadth	Orbits, Nasion	NOR	Distance from nasion (N) to orbitale (Or)
Frontomalare‐nasion length	Midface	FMTN	Distance from frontomalare (Fmt) to nasion (N)
Frontomalare‐nasospinale	Midface	FMTNS	Distance from frontomalaretemporale (Fmt) to nasospinale (Ns)
Malar height	Midface	MAH	Distance from the most inferior point on the lower border of the orbit (Or) to the most superior point on the inferior border of the zygomatic (Z)
Mastoid length	Temporal	MPL	Distance from porion (Po) to mastoidale (Ms)
Mastoid breadth	Temporal	MPB	Distance from the posterior border of the external auditory meatus (Pem) to the most anterior point along the posterior margin of the mastoid process (Pm)
Mastoidale‐asterion	Temporal	MSAST	Distance from mastoidale (Ms) to asterion (Ast)
Occipital condyle length	Base	OCL	Distance from the most anterior (Aoc) to the most posterior (Poc) point on the occipital condyles
Opisthion‐porion length	Base	OPO	Distance from the opisthion (O) to porion (Po)
Basion‐porion length	Base	BAPO	Distance from basion (Ba) to porion (Po)
Nasion‐mastoidale	Nasion	NMS	Distance from nasion (N) to mastoidale (Ms)
Nasion‐alare	Nasion, Midface	NAL	Distance from nasion (N) to alare (Al)
Nasospinale‐alare	Nasion, Midface	NAAL	Distance from nasospinale (Ns) to alare (Al)

**TABLE 3:** Definitions of the FA17 matrices (Harripershad et al., 2024).

Indices	Definition
FA17	Average of all FA8 values
FA17_Orbital	Average of FA8_OBB, FA8_OBH, and FA8_NOR
FA17_Face	Average of FA8_FMTN, FA8_FMTNS, FA8_MAH, FA8_NAL, and FA8_NAAL
FA17_Temporal	Average of FA8_MPL, FA8_MPB, FA8_MSAST, and FA8_NMS
FA17_Base	Average of FA8_OCL, FA8_OPO, and FA8_BAPO

Formula 1:

$$\text{FA8}=|\text{In}\left(R_{d}/L_{d}\right)|.$$

FA8: the difference between the natural logs (ln) of the left and right measurements of a trait (*L*_*d*_ and *R*_*d*_, respectively), in absolute values.

Formula 2:

$$\mathrm{FA}17=\sum |\text{In}\left(R_{d}/L_{d}\right)|/T.$$

FA17: the average FA values computed from the FA8 values for multiple traits and combinations. *T* is the total number of traits per index.

### 2.3 Data analysis

All statistical analyses were conducted in the R software and RStudio environment (RStudio Team, 2020).

#### 2.3.1 Repeatability testing

As the following study focuses on asymmetry, repeatability needs to be thoroughly tested from different aspects, inclusive of: (1) positioning of the landmarks, (2) technical error of measurement, and (3) repeatability of the between‐sides variation. The three protocols followed are detailed in Harripershad et al. (2024). The first protocol assessed the reproducibility (observer agreement) of the landmark positioning, calculated using the dispersion error (in mm) for each landmark and individual on 15 scans. The second protocol was based on the technical error of measurement (TEM) and the relative TEM (%TEM) to assess observer error between left and right traits. The third protocol, using Procrustes ANOVAs, quantifies the variation among repeated measurements (or measurement error) and is calculated with the intra‐observer landmark coordinates.

#### 2.3.2 ILDs and FA indices

Descriptive statistics were calculated to summarise and describe the main features of the dataset for asymmetrical linear distances (ILDs) and FA values (Trochim, 2020). Normality testing was conducted using Shapiro–Wilk tests on the ILDs and on the FA indices. As the results indicated non‐normal distributions, non‐parametric Wilcoxon signed‐rank tests for paired samples were chosen and performed between each corresponding left and right distance (e.g., left, and right OBH) to evaluate asymmetry. Asymmetrical differences between the demographic groups (e.g., sex and population affinity) were further investigated for the ILDs, as well as for FA8 and FA17, using Mann–Whitney–Wilcoxon rank‐sum tests when two samples were compared (i.e., between sex, or population affinity). Kruskal–Wallis rank‐sum tests (with Bonferroni corrections to avoid familywise error) were done when more than two samples were compared (i.e., sex/population interaction) to ensure that pooling the sample did not mask any asymmetry that may have been present. Post‐hoc tests with Bonferroni corrections were conducted in conjunction with the Kruskal–Wallis tests. *p*‐values and 95% confidence intervals (CIs) were used for interpretation of the results.

#### 2.3.3 Geometric morphometric methods

Lastly, GMM analyses were conducted to assess asymmetrical shape variation in demographic profiles. Geometric morphometrics is a method that focuses on biological shape analysis using Cartesian coordinates (*x*, *y*, *z*) of anatomical landmarks (Mitteroecker & Gunz, 2009; Slice, 2007). A Generalised Procrustes Analysis (GPA) was first performed on the raw Cartesian coordinates of the landmarks of each matrix to obtain orientation‐invariant shape coordinates and to remove the scale and position configuration of landmarks in order to minimise the variance between landmarks among individuals (Dryden & Mardia, 2016). The *plotOutliers* function from the *geomorph* package (Adams et al., 2023; Braken et al., 2021) was used to detect any outliers and remove them when necessary. Then, statistical methods for object symmetry, defined by the presence of corresponding bilateral landmarks around a midline axis of symmetry (e.g., right, and left side of human face) (Klingenberg et al., 2002; Mardia et al., 2000), were implemented as defined by the *bilat.symmetry* function of the *geomorph* package (Adams et al., 2023; Braken et al., 2021). Procrustes two‐way mixed‐model analysis of variances (ANOVAs) were performed to statistically quantify asymmetry within each sex, population affinity, and sex/population. This method allows quantification of the main components of shape variation: (1) the overall variation among individuals in the sample (individual fixed effects: *ind*); (2) the variation among the left and right sides (side fixed effects: *side*), also known as directional asymmetry; (3) the variation due to the interaction between an individual and side (*ind: side*), also known as FA. Finally, the symmetric and asymmetric component coordinates were extracted from the *bilat.symmetry* outputs and were used to illustrate shape variation of each matrix between the different groups analysed.

## 3 RESULTS

### 3.1 Repeatability testing

To ensure precise results and that any measurement differences were due to asymmetry rather than observer error, observer repeatability was tested on craniometric landmark positioning. A measurement error threshold of 2 mm was selected as this is deemed acceptable in forensic anthropology and accounts for typical asymmetric variation found in crania (Choi, 2015; Stull, Kenyhercz, et al., 2014). The detailed results are presented in Harripershad et al. (2024). To summarise, lower mean dispersion values were obtained for intra‐observer compared with inter‐observer mean values. All craniometric landmarks presented with an intra‐observer measurement error of less than 2 mm (mean ± SD = 0.7 ± 0.53 mm, range = 0.28–1.43 mm), showing high intra‐observer reproducibility. For inter‐observer repeatability, the majority of the craniometric landmarks presented with a measurement error of less than 2 mm (mean ± SD = 1.3 ± 0.84 mm, range = 0.36–5.04 mm), while three landmarks, the right inferior orbital margin, and the right and left landmarks on the posterior margin of the mastoid, fell above 2 mm.

With regard to TEM and %TEM, intra‐observer variation ranged from 0.45 to 1.48 mm and 0.66% to 4.49%, all within the acceptable range for a skilled observer (Perini et al., 2005). In addition, two‐way ANOVAs were performed to assess measurement error and repeatability between left and right sides. Only one cranial measurement (OBB) showed higher measurement error with no significant difference between sides, so it was excluded from further analysis. This aligns with practices in similar studies, as recommended by Palmer and Strobeck (2002).

Lastly, for GMM analysis, the error component of the Procrustes ANOVAs was assessed, and the level of ME was negligible in comparison with the level of FA, as indicated by the mean square values (MS) (Table 6), suggesting that the shape data are suitable to study FA. Due to limited discrepancies with the intra‐observer dispersion, TEM/%TEM, and the error component of the Procrustes ANOVAs (and with data being collected solely by the principal investigator), all the landmarks and measurements (except for OBB) were considered acceptable and retained for further analysis.

### 3.2 Size variation: ILDs

ILDs were used to quantify size variation of the craniofacial skeleton, and the left and right sides were compared to explore differences attributable to sex and population variation. Mean values were calculated for the left and right ILDs (see Table S1 for descriptive statistics). These results provide information on the mean values for each left and right ILD that describe where the greatest variation lies, so asymmetry could be better quantified within the sample.

**FIGURE 2:**
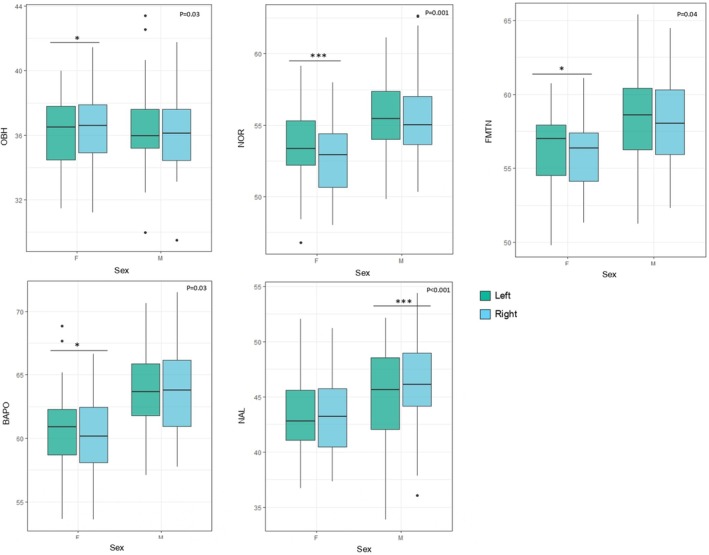
Boxplots illustrating the asymmetry (left side in green, right in blue) in females (F) and males (M), for the statistically significant inter‐landmark distances (in mm) of the Wilcoxon signed‐rank tests (*p* < 0.05). Significance: ***p < 0.001, *p < 0.05. Dots depict outliers. BAPO, basion‐porion length; FMTN, frontomalare‐nasion length; NAL, nasion‐alare; NOR, diagonal orbital breadth; OBH, orbital height.

Wilcoxon signed‐rank tests with 95% CI were performed on all cranial ILDs to test for asymmetry (between the left and right sides) in the entire sample with all the groups pooled, and within subgroups according to sex and population affinity, respectively (Table 4). In the pooled sample, significant statistical left versus right differences were observed for NOR (*p* = 0.001, 95% CI [−0.75, −0.21]) and NAL (*p* < 0.001, 95% CI [0.34, 0.94]), which are variables pertaining to the nasal region. When comparing the left and right distances in males and females, slightly different patterns were observed with significant asymmetrical differences in four ILDs in females (OBH: *p* = 0.03, 95% CI [0.05, 0.78]; NOR: *p* = 0.001, 95% CI [−1.07, −0.31]; FMTN: *p* = 0.04, 95% CI [−0.68, −0.02]; and BAPO: *p* = 0.03, 95% CI [−1.26, −0.10]), and only one in males (NAL: *p* < 0.001, 95% CI [0.51, 1.40]) (refer to Table 4 and Figure 2 for more details). While in females, these variables also pertain largely to the nasal and orbital regions, they include other ILDs than those reported for the pooled sample.

**TABLE 4:** Wilcoxon signed‐rank tests comparing left and right bilateral distances for the pooled sample, per sex (F—females; M—males), population affinity (B—black; W—white) and sex/population subgroups (BF—black female; BM—black male; WF—white female; WM—white male). 95% confidence intervals (CI) are detailed.

	Pooled sample	Sex		Population affinity		Sex/population			
		F	M	B	W	BF	BM	WF	WM
OBH									
*p*‐value	0.44	**0.03**	0.20	**0.03**	0.29	**0.01**	0.78	0.86	0.13
95% CI	[−0.16, 0.34]	**[0.05, 0.78]**	[−0.54, 0.13]	**[0.03, 0.68]**	[−0.55, 0.18]	**[0.26, 1.24]**	[−0.50, 0.46]	[−0.55, 0.57]	[−0.83, 0.16]
NOR									
*p*‐value	**<0.001**	**<0.001**	0.18	**0.02**	**0.02**	0.08	0.14	**<0.001**	0.64
95% CI	**[−0.75, −0.21]**	**[−1.07, −0.31]**	[−0.67, 0.12]	**[−0.78, −0.08]**	**[−1.01, −0.11]**	[−0.99, 0.08]	[−0.84, 0.09]	**[−1.44, −0.35]**	[−0.87, 0.47]
FMTN									
*p*‐value	0.07	**0.04**	0.51	0.12	0.31	0.07	0.73	0.36	0.60
95% CI	[−0.50, 0.02]	**[−0.68, −0.02]**	[−0.55, 0.27]	[−0.71, 0.08]	[−0.55, 0.19]	[−0.97, 0.05]	[−0.71, 0.45]	[−0.60, 0.25]	[−0.84, 0.48]
FMTNS									
*p*‐value	0.14	0.43	0.20	**0.01**	0.72	**0.03**	0.10	0.41	0.82
95% CI	[−0.51, 0.07]	[−0.55, 0.23]	[0.69, 0.16]	**[−0.88, −0.12]**	[−0.36, 0.55]	**[−1.11, −0.06]**	[−1.00, 1.14]	[−0.34, 0.82]	[−0.76, 0.65]
MAH									
*p*‐value	0.34	0.05	0.60	0.13	0.84	**0.03**	1.00	0.50	0.42
95% CI	[−0.16, 0.43]	[0.00, 0.72]	[−0.58, 0.35]	[−0.12, 0.75]	[−0.47, 0.34]	**[0.03, 1.16]**	[−0.53, 0.68]	[−0.38, 0.58]	[−0.92, 0.41]
MPL									
*p*‐value	0.37	0.99	0.20	0.60	0.45	1.00	0.42	0.97	0.26
95% CI	[−0.53, 0.37]	[−0.49, 0.51]	[−0.96, 0.20]	[−0.70, 0.35]	[−0.69, 0.31]	[−0.58, 0.53]	[−1.46, 0.61]	[−0.74, 1.00]	[−1.03, 0.31]
MPB									
*p*‐value	0.98	0.85	0.93	0.62	0.63	0.25	0.62	0.15	0.52
95% CI	[−0.66, 0.67]	[−0.74, 0.90]	[−1.21, 1.24]	[−1.20, 0.82]	[−0.74, 1.16]	[−1.66, 0.39]	[−1.51, 3.73]	[−0.36, 2.23]	[−1.91, 1.05]
MSAST									
*p*‐value	0.44	0.16	0.80	**0.01**	0.19	**0.04**	0.10	0.93	0.06
95% CI	[−0.46, 0.97]	[−0.23, 1.46]	[−1.51, 1.04]	**[0.30, 1.91]**	[−2.09, 0.41]	**[0.06, 2.11]**	[−0.25, 2.40]	[−1.44, 1.44]	[−4.15, 0.16]
OCL									
*p*‐value	0.15	0.10	0.69	0.22	0.39	0.28	0.44	0.16	0.82
95% CI	[−0.62, 0.10]	[−0.91, 0.08]	[−0.68, 0.48]	[−0.79, 0.16]	[−0.80, 0.35]	[−0.75, 0.24]	[−1.23, 0.46]	[−1.60, 0.18]	[−0.59, 1.03]
OPO									
*p*‐value	0.63	0.85	0.32	0.82	0.55	0.78	0.47	0.92	0.42
95% CI	[−0.29, 0.48]	[−0.66, 0.52]	[−0.28, 0.80]	[−0.48, 0.54]	[−0.42, 0.79]	[−1.03, 0.67]	[−0.39, 0.79]	[−0.78, 0.82]	[−0.58, 1.35]
BAPO									
*p*‐value	0.09	**0.03**	0.82	**0.02**	0.87	0.18	**0.04**	0.07	0.28
95% CI	[−0.81, 0.06]	**[−1.26, −0.10]**	[−0.67, 0.57]	**[−1.22, −0.13]**	[−0.71, 0.62]	[−1.62, 0.33]	**[−1.28, −0.04**]	[−1.52, 0.03]	[−0.23, 1.61]
NMS									
*p*‐value	0.72	0.26	0.10	0.58	0.31	0.44	0.92	0.43	**0.03**
95% CI	[−0.58, 0.42]	[−0.25, 1.08]	[−1.24, 0.12]	[−0.47, 0.89]	[−1.13, 0.36]	[−0.56, 1.40]	[−0.98, 0.95]	[−0.55, 1.49]	**[−2.10, −0.19]**
NAL									
*p*‐value	**<0.001**	0.13	**<0.001**	**<0.001**	**0.01**	0.23	**<0.001**	0.28	**0.01**
95% CI	**[0.34, 0.94]**	[−0.09, 0.78]	**[0.51, 1.40]**	**[0.27, 1.11]**	**[0.20, 1.05]**	[−0.22, 0.97]	**[0.35, 1.62]**	[−0.33, 0.91]	**[0.20, 1.60]**
NAAL									
*p*‐value	0.92	0.31	0.29	0.46	0.55	0.90	0.30	0.19	0.73
95% CI	[−0.20, 0.25]	[−0.16, 0.48]	[−0.62, 0.19]	[−0.46, 0.23]	[−0.26, 0.48]	[−0.42, 0.44]	[−0.86, 0.24]	[−0.18, 0.79]	[−0.76, 0.43]

*Note*: Bold indicates statistically significant differences (*p* < 0.05).

Abbreviations: BAPO, basion‐porion length; FMTN, frontomalare‐nasion length; FMTNS, frontomalare‐nasospinale; MAH, malar height; MPB, mastoid breadth; MPL, mastoid length; MSAST, mastoidale‐asterion; NAAL, nasospinale‐alare; NAL, nasion‐alare; NMS, nasion‐mastoidale; NOR, diagonal orbital breadth; OBH, orbital height; OCL, occipital condyle length; OPO, opisthion‐porion length.

**FIGURE 3:**
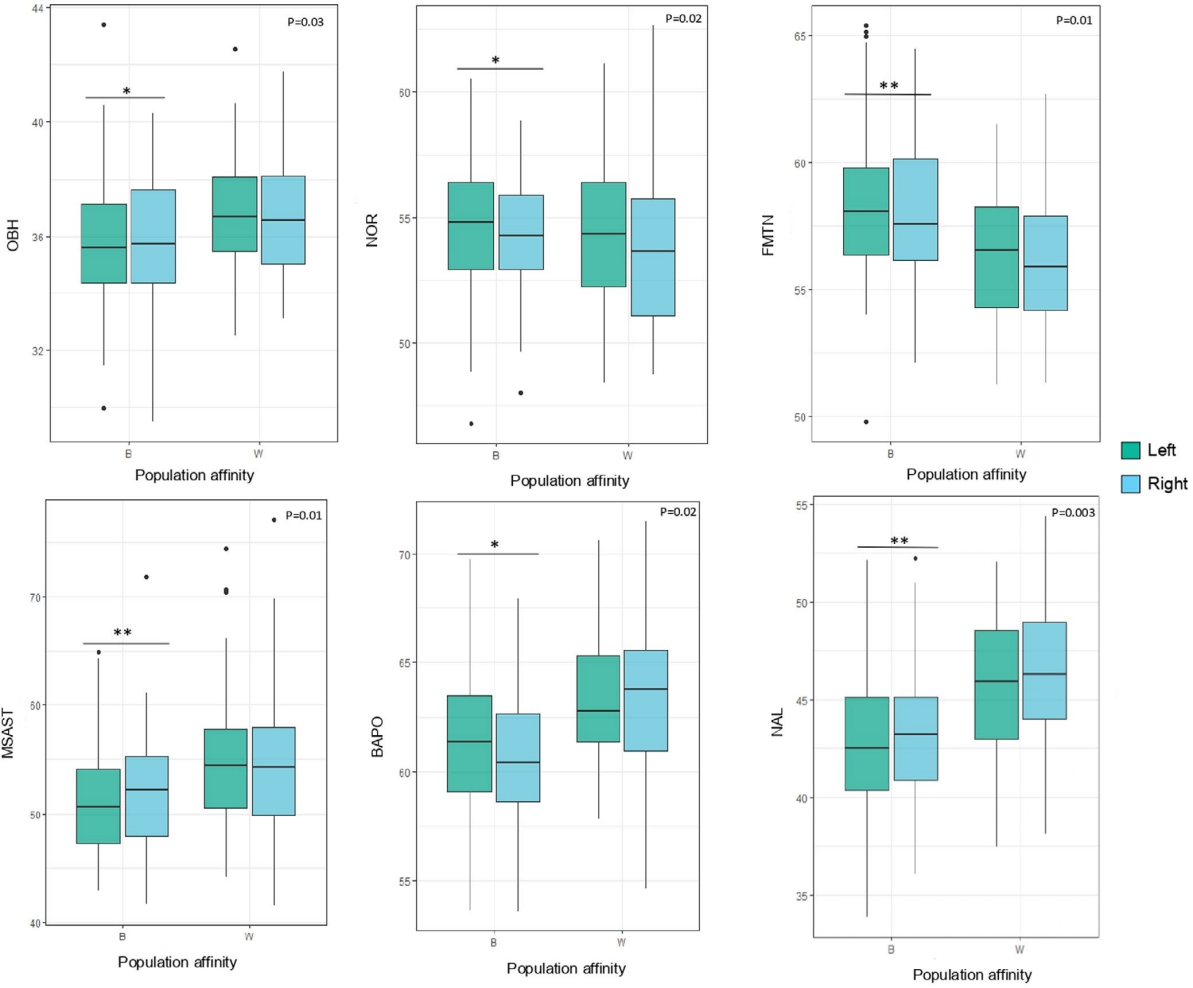
Boxplots illustrating the asymmetry (left side in green, right in blue) in black (B) and white (W) South Africans for the statistically significant inter‐landmark distances (in mm) of the Wilcoxon signed‐rank tests (*p* < 0.05). Significance: **p < 0.01, *p < 0.05. Dots depict outliers. BAPO, basion‐porion length; FMTNS, frontomalare‐nasospinale; MSAST, mastoidale‐asterion; NAL, nasion‐alare; NOR, diagonal orbital breadth; OBH, orbital height.

Regarding population affinity, both black and white South Africans demonstrated similar results to the pooled sample, in which statistically significant asymmetrical differences were detected for OBH, NOR, FMTNS, MSAST, BAPO, and NAL (*p* < 0.05) (refer to Table 4 and Figure 3). White South Africans demonstrated significant left versus right differences for NOR (*p* = 0.02, 95% CI [−1.01, −0.11]) and NAL (*p* = 0.01, 95% CI [0.20, 1.05]) (similarly as in the pooled sample); while black South Africans displayed significant differences for OBH (*p* = 0.03, 95% CI [0.03, 0.68]), NOR (*p* = 0.02, 95% CI [−0.78, −0.08]), FMTNS (*p* = 0.01, 95% CI [−0.88, −0.12]), MSAST (*p* = 0.01, 95% CI [0.30, 1.91]), BAPO (*p* = 0.02, 95% CI [−1.22, −0.13]) and NAL (*p* = 0.003, 95% CI [0.27, 1.11]), which pertains to other cranial areas in addition to the orbital and nasal regions. Lastly, black South Africans expressed higher levels of asymmetrical variation with significant differences between left and right sides in six ILDs compared with only three in white South Africans.

**FIGURE 4:**
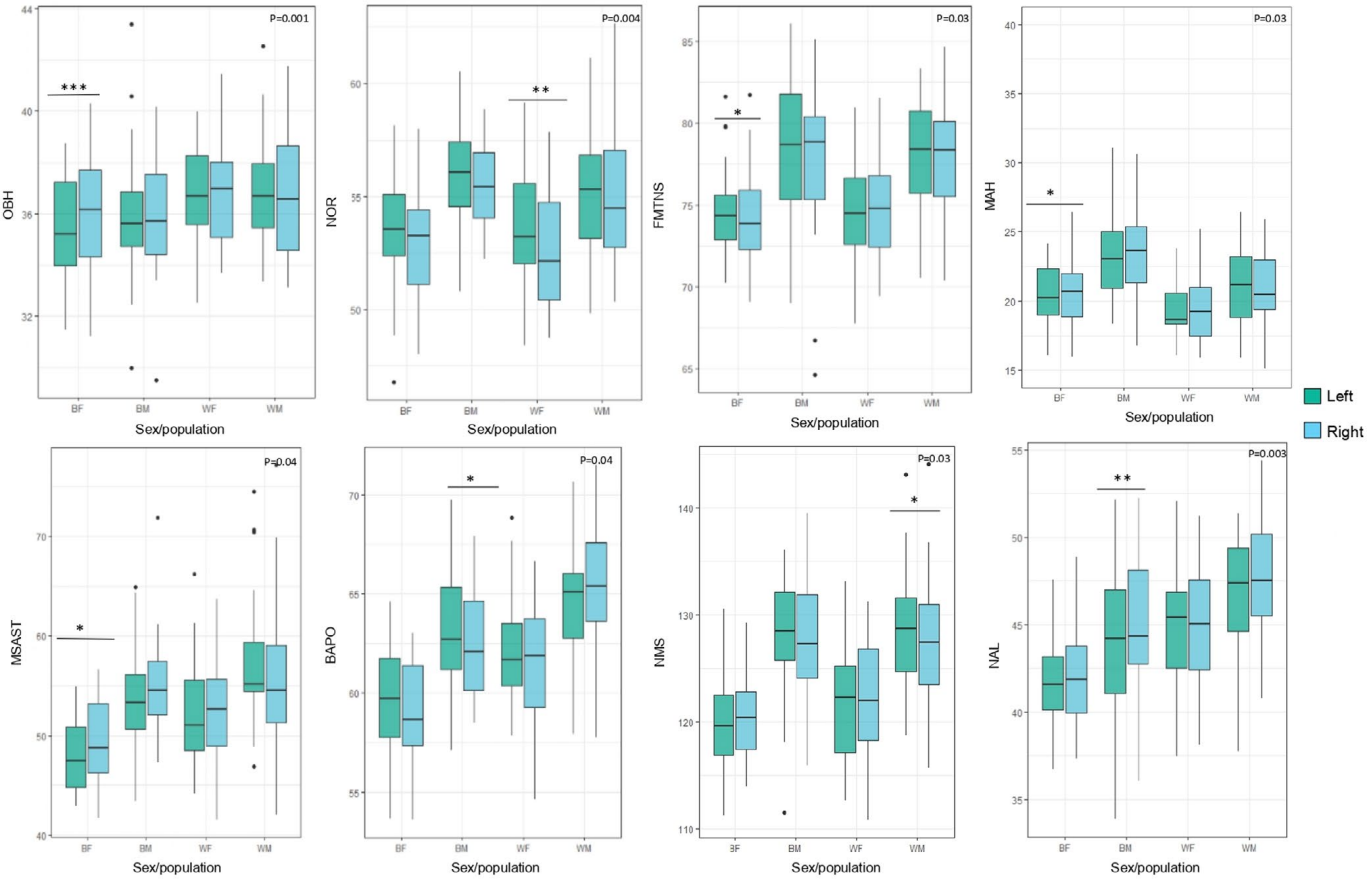
Boxplots illustrating the asymmetry (left side in green, right in blue) in sex/population subgroups (BF—black females; BM—black males; WF—white females; and WM—white males) for the statistically significant inter‐landmark distances (in mm) of the Wilcoxon signed‐rank tests (*p* < 0.05). Significance: ***p < 0.001, **p < 0.01, *p < 0.05. Dots depict outliers. BAPO, basion‐porion length; FMTNS, frontomalare‐nasospinale; MAH, malar height; MSAST, mastoidale‐asterion; NAL, nasion‐alare; NMS, nasion‐mastoidale; NOR, diagonal orbital breadth; OBH, orbital height.

When assessing each sex and population subgroup simultaneously, significant asymmetrical differences were noted for OBH, NOR FMTNS, MAH, MSAST, BAPO, NMS, and NAL (*p* < 0.05) (refer to Table 4 and Figure 4 for more details). Overall, black South African females showed higher levels of significant asymmetrical variation among all subgroups in four significant distances (OBH, FMTNS, MAH, MSAST), while only two were reported for black and white South African males and one for white South African females.

### 3.3 Size variation: FA indices

After assessing the raw ILDs, mean values of FA8 and FA17 indices were calculated (descriptive statistics available in Table 5).

**TABLE 5:** Descriptive statistics for FA8 and FA17 values per sex (F—females; M—males), and in the entire sample.

	F (*N* = 57)		M (*N* = 58)		Total (*N* = 115)	
	Mean (SD)	Min–max	Mean (SD)	Min–max	Mean (SD)	Min–max
FA8_OBH	0.03 (0.02)	0.00–0.09	0.03 (0.02)	0.00–0.08	0.03 (0.02)	0.00–0.09
FA8_NOR	0.02 (0.02)	0.00–0.08	0.02 (0.02)	0.00–0.07	0.02 (0.02)	0.00–0.08
FA8_FMTN	0.02 (0.01)	0.00–0.06	0.02 (0.02)	0.00–0.07	0.02 (0.02)	0.00–0.07
FA8_FMTNS	0.02 (0.01)	0.00–0.04	0.02 (0.01)	0.00–0.07	0.02 (0.01)	0.00–0.07
FA8_MAH	0.06 (0.05)	0.00–0.17	0.06 (0.05)	0.00–0.23	0.06 (0.05)	0.00–0.23
FA8_MPL	0.05 (0.06)	0.00–0.14	0.05 (0.04)	0.00–0.15	0.05 (0.04)	0.00–0.15
FA8_MPB	0.08 (0.07)	0.00–0.42	0.11 (0.10)	0.00–0.51	0.10 (0.09)	0.00–0.51
FA8_MSAST	0.06 (0.05)	0.00–0.32	0.07 (0.08)	0.00–0.37	0.07 (0.07)	0.00–0.37
FA8_OCL	0.07 (0.07)	0.01–0.28	0.08 (0.06)	0.00–0.24	0.07 (0.06)	0.00–0.28
FA8_OPO	0.02 (0.02)	0.00–0.09	0.02 (0.02)	0.00–0.07	0.02 (0.02)	0.00–0.09
FA8_BAPO	0.03 (0.03)	0.00–0.12	0.03 (0.02)	0.00–0.09	0.03 (0.03)	0.00–0.12
FA8_NMS	0.02 (0.01)	0.00–0.05	0.02 (0.01)	0.00–0.05	0.02 (0.01)	0.00–0.05
FA8_NAL	0.03 (0.02)	0.00–0.08	0.03 (0.03)	0.00–0.10	0.03 (0.02)	0.00–0.10
FA8_NAAL	0.06 (0.06)	0.01–0.36	0.07 (0.06)	0.00–0.35	0.07 (0.06)	0.00–0.36
FA17	0.04 (0.01)	0.02–0.07	0.04 (0.01)	0.02–0.07	0.04 (0.01)	0.02–0.07
FA17_Orbital	0.026 (0.01)	0.00–0.06	0.03 (0.01)	0.01–0.07	0.03 (0.01)	0.00–0.07
FA17_Face	0.03 (0.02)	0.01–0.08	0.03 (0.02)	0.01–0.09	0.03 (0.02)	0.01–0.09
FA17_Temporal	0.06 (0.04)	0.01–0.23	0.08 (0.05)	0.02–0.27	0.07 (0.04)	0.01–0.27
FA17_Base	0.04 (0.02)	0.01–0.09	0.04 (0.02)	0.01–0.08	0.04 (0.02)	0.01–0.09

Mann–Whitney–Wilcoxon rank‐sum tests revealed no significant differences in asymmetry based on FA indices between the sexes and population groups, as all *p*‐values exceeded 0.05. Similarly, Kruskal–Wallis tests (with Bonferroni corrections), which assessed asymmetry considering sex and population simultaneously, also showed no statistically significant differences.

### 3.4 Shape variation: GMM

**FIGURE 5:**
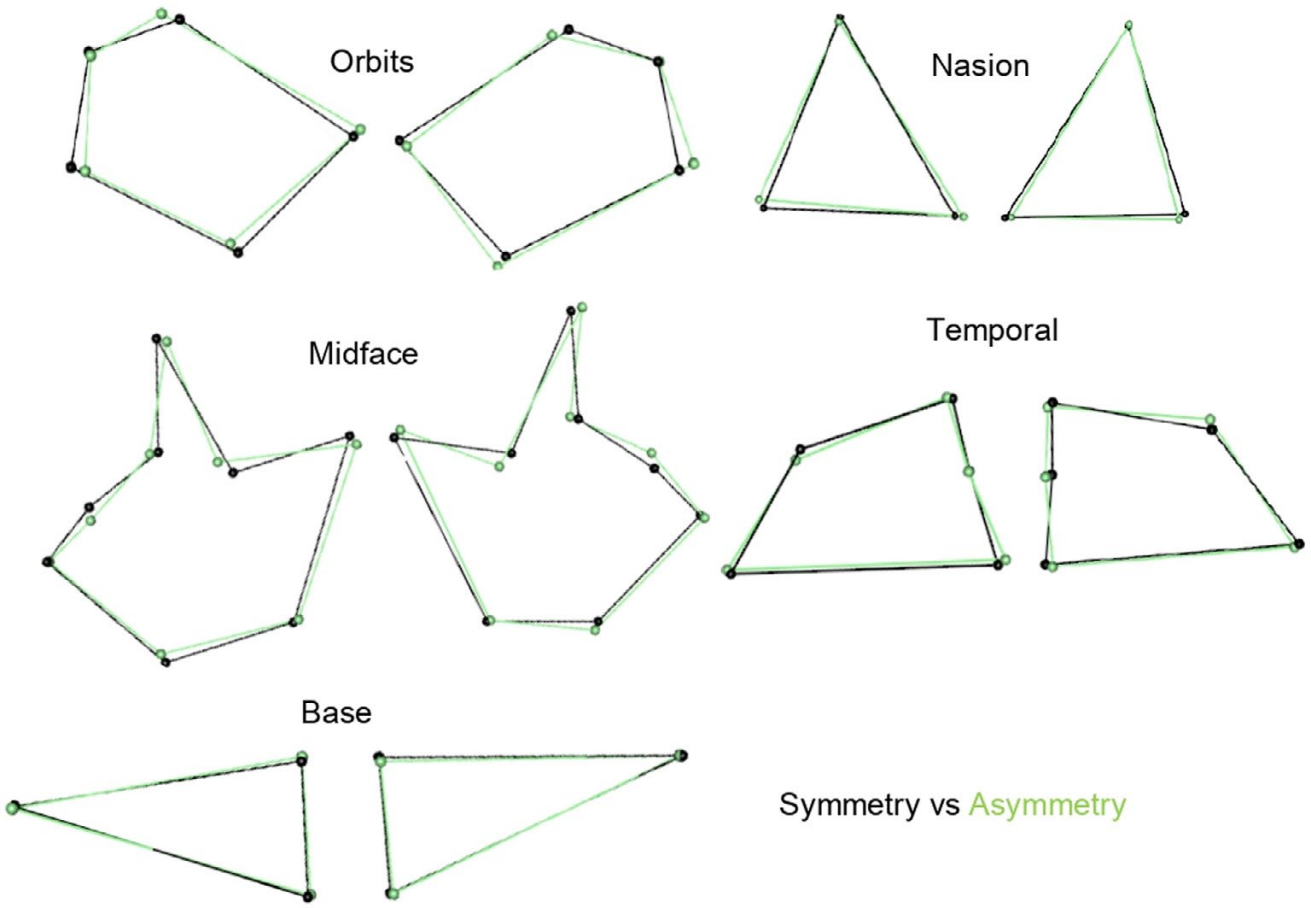
Illustration of symmetrical (black) and asymmetrical (green) shape differences for all geometric morphometric method matrices in the pooled sample.

The analysis of shape demonstrated similar patterns as the ILDs. More specifically, the results for FA for the pooled sample yielded statistically significant shape differences for the nasion, midface, orbits, temporal, and base regions (*p* < 0.05) (Table 6; Figure 5). According to the *F*‐values, the greatest asymmetrical variation was found in the base region, followed by the temporals, nasion, midface, and orbits (Table 6; Figure 5).

**TABLE 6:** Procrustes ANOVA outputs for object asymmetry for the entire sample, per sex (F—females; M—males), population affinity (B—black; W—white) and sex/population groups (BF—black female; BM—black male; WF—white female; WM—white male).

	Base				Midface				Orbits				Nasion				Temporal			
	SS	MS	*F*	Pr (>*F*)	SS	MS	*F*	Pr (>*F*)	SS	MS	*F*	Pr (>*F*)	SS	MS	*F*	Pr (>*F*)	SS	MS	*F*	Pr (>*F*)
Total sample																				
Ind	0.87	0.01	3.37	1.00	0.84	0.01	5.08	1.00	0.65	0.01	4.97	0.95	1.24	0.01	6.49	0.80	0.76	0.01	3.11	1.00
Side (DA)	0.00	0.00	1.63	0.15	0.04	0.04	28.67	**<0.001**	0.05	0.05	39.6	**<0.001**	0.03	0.03	17.34	**<0.001**	0.02	0.02	6.81	**<0.001**
Ind:side (FA)	0.26	0.00	3.70	**<0.001**	0.17	0.00	1.85	**<0.001**	0.13	0.00	1.33	**0.01**	0.19	0.00	2.05	**<0.001**	0.24	0.00	3.64	**<0.001**
Error	0.02	0.00			0.02	0.00			0.02	0.00			0.03	0.00			0.02	0.00		
F																				
Ind	0.42	0.01	3.01	0.99	0.41	0.01	5.20	0.91	0.33	0.01	5.27	0.58	0.58	0.01	6.83	0.44	0.36	0.01	3.60	1.00
Side (DA)	0.01	0.01	2.25	0.05	0.03	0.03	19.26	**<0.001**	0.03	0.03	23.40	**<0.001**	0.02	0.02	13.63	**<0.001**	0.01	0.01	4.61	**<0.001**
Ind:side (FA)	0.14	0.00	4.24	**<0.001**	0.07	0.00	2.22	**<0.001**	0.06	0.00	1.32	**0.02**	0.09	0.00	1.75	**<0.001**	0.01	0.00	4.74	**<0.001**
Error	0.01	0.00			0.01	0.00			0.01	0.00			0.01	0.00			0.01	0.00		
M																				
Ind	0.44	0.01	3.89	0.99	0.40	0.01	4.82	0.99	0.29	0.01	4.41	0.99	0.60	0.01	6.06	0.91	0.37	0.01	2.69	1.00
Side (DA)	0.00	0.00	0.83	0.53	0.02	0.02	11.58	**<0.001**	0.02	0.02	17.70	**<0.001**	0.01	0.01	7.16	**<0.001**	0.01	0.01	3.60	**0.01**
Ind:side (FA)	0.11	0.00	4.24	**<0.001**	0.08	0.00	1.24	0.17	0.07	0.00	1.29	0.09	0.01	0.00	2.73	**<0.001**	0.14	0.00	2.14	**0.01**
Error	0.01	0.00			0.01	0.00			0.01	0.00			0.01	0.00			0.01	0.00		
B																				
Ind	0.46	0.01	3.74	0.97	0.41	0.01	4.99	0.93	0.29	0.01	4.69	0.65	0.54	0.01	6.36	0.56	0.35	0.01	3.53	0.98
Side (DA)	0.00	0.00	1.86	0.09	0.02	0.00	14.46	**<0.001**	0.02	0.02	19.08	**<0.001**	0.02	0.02	11.29	**<0.001**	0.01	0.001	5.02	**<0.001**
Ind:side (FA)	0.12	0.00	4.24	**<0.001**	0.08	0.00	2.27	**<0.001**	0.06	0.00	1.25	**0.04**	0.09	0.00	1.97	**<0.001**	0.10	0.00	3.23	**<0.001**
Error	0.01	0.00			0.01	0.00			0.02	0.00			0.02	0.00			0.01	0.00		
W																				
Ind	0.38	0.01	2.90	1.00	0.32	0.01	4.00	1.00	0.26	0.01	4.00	1.00	0.42	0.01	4.18	0.97	0.38	0.01	2.83	1.00
Side (DA)	0.00	0.00	1.12	0.34	0.02	0.02	15.91	**<0.001**	0.03	0.03	22.29	**<0.001**	0.01	0.01	7.81	**<0.001**	0.01	0.01	5.81	**<0.001**
Ind:side (FA)	0.13	0.00	4.24	**<0.001**	0.08	0.00	1.20	0.19	0.07	0.00	1.36	0.06	0.10	0.00	1.87	**0.01**	0.13	0.00	3.52	**<0.001**
Error	0.00	0.00			0.01	0.00			0.01	0.00			0.01	0.00			0.01	0.00		
BF																				
Ind	0.22	0.01	3.25	0.89	0.21	0.01	4.75	0.74	0.15	0.01	5.24	0.21	0.29	0.01	6.43	0.33	0.17	0.01	4.22	0.84
Side (DA)	0.00	0.00	1.50	0.20	0.02	0.02	9.93	**<0.001**	0.01	0.01	14.34	**<0.001**	0.01	0.01	7.71	**<0.001**	0.01	0.01	3.11	**0.01**
Ind:side (FA)	0.07	0.00	3.75	**<0.001**	0.04	0.00	2.41	**<0.001**	0.03	0.00	1.22	0.10	0.05	0.00	1.90	**<0.001**	0.04	0.00	3.97	**<0.001**
Error	0.01	0.00			0.01	0.00			0.01	0.00			0.01	0.00			0.01	0.00		
BM																				
Ind	0.23	0.01	4.40	0.92	0.18	0.01	4.93	0.95	0.12	0.01	3.93	0.95	0.24	0.01	6.09	0.84	0.17	0.01	2.86	0.98
Side (DA)	0.00	0.00	0.73	0.62	0.01	0.01	4.94	**<0.001**	0.01	0.01	6.18	**0.02**	0.01	0.01	4.18	**0.01**	0.01	0.01	2.55	0.05
Ind:side (FA)	0.05	0.00	3.03	**<0.001**	0.04	0.00	2.01	**0.01**	0.03	0.00	1.07	0.44	0.04	0.00	2.53	0.01	0.06	0.00	1.15	0.18
Error	0.00	0.00			0.00	0.00			0.00	0.00			0.00	0.00			0.01	0.00		
WF																				
Ind	0.18	0.00	2.53	0.99	0.14	0.01	4.02	1.00	0.13	0.01	3.98	0.96	0.18	0.01	4.47	0.86	0.18	0.01	3.13	1.00
Side (DA)	0.00	0.00	1.12	0.38	0.02	0.02	0.08	**0.01**	0.01	0.01	11.35	**0.01**	0.01	0.01	6.76	**<0.001**	0.01	0.01	3.28	**0.01**
Ind:side (FA)	0.07	0.00	6.72	**<0.001**	0.03	0.00	1.69	**0.01**	0.03	0.00	1.17	0.33	0.04	0.00	1.23	0.33	0.06	0.00	4.73	**<0.001**
Error	0.00	0.00			0.00	0.00			0.00	0.00			0.01	0.00			0.02	0.00		
WM																				
Ind	0.20	0.01	3.49	0.95	0.17	0.01	3.86	0.98	0.12	0.00	3.69	1.00	0.24	0.01	4.12	0.94	0.19	0.01	2.67	0.99
Side (DA)	0.00	0.00	2.32	0.07	0.01	0.01	7.46	**<0.001**	0.02	0.02	12.46	**0.01**	0.01	0.01	4.17	**0.01**	0.01	0.01	4.51	**0.02**
Ind:side (FA)	0.05	0.00	3.04	**<0.001**	0.04	0.00	0.93	0.64	0.03	0.00	1.62	**0.03**	0.06	0.00	2.85	**0.01**	0.07	0.00	2.83	**0.01**
Error	0.00	0.00			0.01	0.00			0.00	0.00			0.00	0.00			0.00	0.00		

*Note*: Bold indicates significance (*p* < 0.05). Ind: individual effects representing the overall variation; side (DA): measure of directional asymmetry (DA); ind:side (FA): measure of fluctuating asymmetry (FA); error: residual measurement error calculated from the variation among repeated measurements.

Abbreviations: *F*, *F* statistic of Procrustes ANOVA; MS, mean squares; Pr(>*F*), *p*‐value; SS, sum of squares.

**FIGURE 6:**
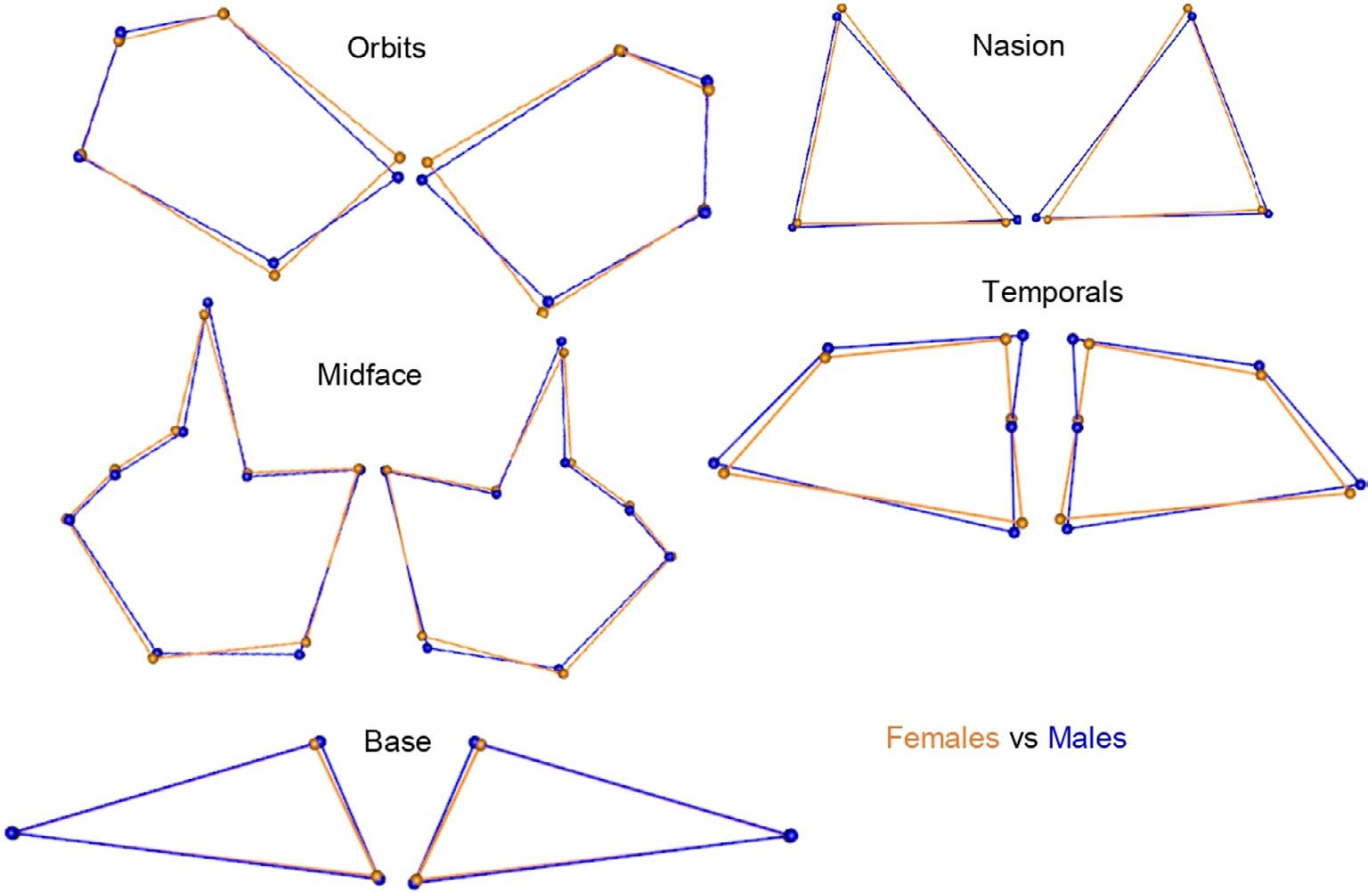
Illustration of asymmetrical shape differences between South African females (orange) and males (blue) for all geometric morphometric method matrices.

**FIGURE 7:**
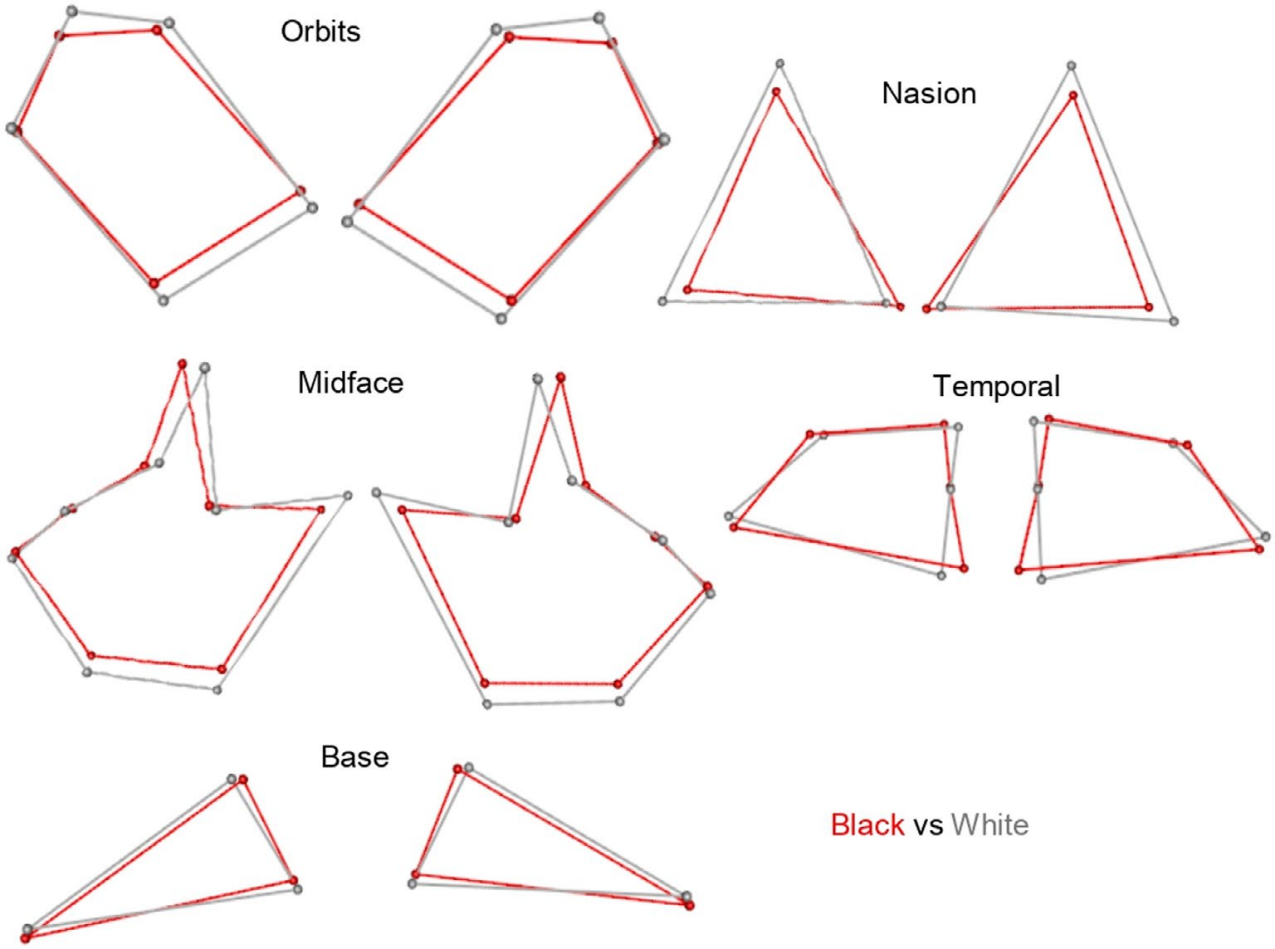
Illustration of asymmetrical shape differences between black South African (red) and white South African (grey) for all geometric morphometric method matrices.

Procrustes ANOVAs were also conducted for each sex, population affinity, and sex and population simultaneously. The FA component demonstrated statistically significant shape differences in both males and females for the nasion, temporal, and base regions (*p* < 0.05) (Table 6; Figure 6). For the midface and orbits, only females demonstrated statistically significant asymmetric shape differences. The greatest variation was observed in females compared with males for almost all regions (greater *F*‐value and significant *p*‐values in females compared with males); however, it is worth noting that males showed greater variation in the nasal region (greatest *F*‐value) (*p* < 0.05) (see Table 6; Figure 6). The FA results for population affinity showed statistically significant shape differences in both black and white South Africans for the nasion, temporal, and base regions (*p* < 0.05). Only black South Africans showed statistically significant shape differences in the midface and orbits (*p* < 0.05) (Table 6; Figure 7). The greatest variation was observed in black South Africans compared with white South Africans for all regions except the base and temporal regions (greatest *F*‐value and significant *p*‐values in black South Africans) (Table 6; Figure 7). However, upon closer examination among sex/population subgroups, black South African females showed the overall greatest variation for FA for all regions except the orbits and base (greatest *F*‐values and significant *p*‐values) (*p* < 0.05) (Table 6), even if the variation in the orbits had a relatively low *F*‐value. It is also worth noting that white South African males showed greater variation in the base region (greatest *F*‐value) (*p* < 0.05).

## 4 DISCUSSION

Skeletal asymmetry is often discussed in the literature, notably to better understand the overall health, lifestyle (e.g., diet, hygiene, behavioral patterns), and diseases in a sample (DeLeon, 2007; Graham & Özener, 2016; Hagg et al., 2017; Harripershad et al., 2024; Kujanová et al., 2008). Asymmetry can affect different populations in various ways, depending on the stressors placed upon them. For example, previous research has shown that past populations in Europe experienced various levels of dental FA brought upon by environmental and genetic factors (Milella et al., 2018). While another study compared dental FA in black South Africans to Paraguayan Indians living under similar environmental stressors but found levels of FA to be higher in the South African group (Kieser & Groeneveld, 1988). As such, studying cranial FA in different populations can provide invaluable insights on how stressors may have implications on the overall skeletal health of a sample. This research is the first study to assess craniofacial FA in a recent South African sample, using 3D imaging and a combination of size and shape variation analyses. The following sections will discuss the prevalence of craniofacial FA within and between populations and sexes, the general trends and patterns observed within the cranium for ILDs, FA indices and shape variations, as well as the overall implications of asymmetry in the South African population.

### 4.1 FA and biological parameters

According to the ILDs, the levels of craniofacial asymmetry differences between the population groups indicated that population‐level trait variation exists. While the GMM results indicate that black South Africans expressed higher levels of craniofacial FA compared with white South Africans, levels of FA noted by GMM are assumed to be in part due to the effect of apartheid on the South African population. This is consistent with our first suggested hypothesis. Furthermore, similar findings were reported by Kieser and Groeneveld (1988) when assessing dental FA in the South African population. The authors attributed the high degree of FA observed in the black South African population to the legacy of apartheid, which resulted in prolonged and persistent unequal living conditions between population groups. Black South Africans were subjected to harsh living conditions and poverty, while white South Africans were typically of higher SES and lived wealthier lifestyles due to the implementation of apartheid racial laws (Alblas et al., 2018; Fogel, 2019; Liebenberg et al., 2015; Linford, 2017; Stullet, Tise, al., 2014). Thus, black South Africans experienced numerous stressors such as poor nutrition, limited resources leading to poorer sanitation and susceptibility to illness, which had dire implications on skeletal growth and formation (Harripershad et al., 2024; Kieser & Groeneveld, 1988; Liebenberg et al., 2015). Even though the apartheid regime ended in 1994, black South African individuals continued to be affected despite the onset of democracy (Mariotti & Fourie, 2014). As a consequence of population history and low SES, many black South Africans have experienced stressors (environmental, genetic, and nutritional stress) that may contribute to the magnitude of FA on the skeleton. On the other hand, while differences were noted for GMM, no statistically significant levels of FA were noted for FA indices between the populations (refer to Harripershad et al., 2024 for a more detailed analysis of FA indices). A possible explanation for the lack of statistically significant FA indices may be due to the fact that FA indices are unable to detect subtle FA differences at a population level. Indeed, in addition, the sample size of this study may reduce the ability of FA indices to detect statistically significant differences in FA.

Within the sample, according to the ILD and GMM analyses, females expressed the most craniofacial variation and FA overall compared with males, rejecting our second hypothesis. This is similar to other populations studied in the literature, such as in Hagg et al. (2017) and Kujanová et al. (2008). Both studies found females to be more asymmetrical than males in archaeological European populations, even if it was not significant in the studies. While Özener and Fink (2010) did not find any significant differences between males and females living in good conditions, they observed that males living in poor conditions were the most asymmetric, and that males from a mixed‐ethnic sample showed significant asymmetry. Asymmetry expression is variable and dependent on the population studied as well as the environmental stressors placed on the populations. While females are often thought to be the least asymmetrical, it should be noted that if females experienced severe stressful conditions during their development, their buffering system fails under extreme stress and becomes inefficient (Graham & Özener, 2016). With generational poverty, which is assumed to likely be the case for the present sample, black South African females may have been unable to buffer negative stressors. In addition, cultural implication may also have an influence, as within South African communities male children are often prioritised for resources leading to females being under higher levels of stress (Coovadia et al., 2009). As such, it is not surprising that black South African females would exhibit the most craniofacial variation and FA overall due to their population history, and stressors placed upon them, which was further confirmed by the ILD and GMM analyses.

### 4.2 Asymmetrical trends in the cranium

General trends in the cranium, for both ILDs (NOR, NAL) and GMM, indicated that the nasal regions exhibited high levels of cranial asymmetry and craniofacial FA in the South African population. GMM indicated that the temporal, midface, and base regions also exhibited high levels of FA. FA in the temporal bones and base is consistent with the literature (DeLeon, 2007; Gawlikowska et al., 2007; Hagg et al., 2017; Storm & Knüsel, 2005). FA in the temporal region is largely influenced by inherited genetic patterns that are neutral (i.e., not affected by natural selection) (von Cramon‐Taubadel, 2014). However, functional areas on the face and base are shaped by environmental factors and follow a non‐neutral pattern of variation (Martínez‐Abadías et al., 2009). Although the nose is under strict genetic control, it should be noted that nasal breadth does not show an additive genetic variance. Additionally, FA often noted in the facial and basal regions tends to be linked to functional asymmetry of the brain and usually increases with nutritional and physiological stress (Ulijaszek & Mascie‐Taylor, 1994). The face is also extremely sensitive to non‐genetic factors (Siebert & Swindler, 2002). Craniofacial traits can exhibit genetic variation, but factors such as sex, year of birth, population, environment, growth, and development can affect the expression of facial traits (Martínez‐Abadías et al., 2009; Sajid et al., 2018). Consequently, population, sex, and the environment should be considered when examining the expression of FA in facial traits (Martínez‐Abadías et al., 2009). The expression of FA in traits and matrices is highly dependent on the strength and ability of canalisation; that is, certain traits may buffer stressors differently. Therefore, the results of the present study confirm the literature, with a fluctuation of the expression of FA across traits and matrices (Blackburn, 2011; Hagg et al., 2017; Møller & Swaddle, 1997; Storm, 2009). Due to the population history and environmental conditions in the South African context, important asymmetrical influences in different regions of the crania were expected. It is worth noting that GMM detected more subtle FA changes in shape compared with FA indices, allowing us to conclude that there is more FA in shape variation than in size variation, as reported in other studies (Benítez et al., 2020; Klingenberg, 2015; Schlager, 2013). However, *p*‐values calculated in this study are limited in the information they can provide; even though significant differences were noted between the left and right sides for ILD and GMM, there might not be any practical applicability for the asymmetry observed.

Given the areas (nasal, temporal, midface, and base region) affected by craniofacial asymmetry and FA in the cranium, it is important to note the implications these traits may have on the biological profile estimates in forensic anthropology. High levels of FA in craniofacial regions may impact the assessment of traits often used in sex and population affinity estimation, affecting biological profile estimates. While the potential influence of asymmetry on estimating the biological profile may be minimal when employing craniometric data, anthropologists should always consider its presence and be cognizant of the sides and elements being used in methods on an individual basis. Given that this study found higher levels of FA in shape than in size of traits, caution should be employed for morphological scoring methodologies that focus on the shape of traits. For example, in terms of morphological scoring (specifically methods developed by Walker (2008) and Hefner (2009) for sex and population affinity estimation), both left and right sides should be included during the process, especially when scoring methods include the mastoids, the midface, and nasal region, as this may potentially decrease the reliability of biological profile estimates focusing on sex and population affinity.

### 4.3 Consideration of secular trends

As this sample spans over 100 years, it is important to consider whether secular trends may have influenced asymmetrical patterns. While our data set would not allow for direct analysis of the trends, it is important to acknowledge the potential impact it could have on our results. Secular trends refer to temporal changes in biological traits across generations (Myburgh et al., 2017; Tobias, 1985). In the context of FA, secular trends can indicate shifts in developmental stability in response to environmental and socioeconomic factors. Over time, favorable environmental and socioeconomic conditions can result in lowered FA values while unfavorable conditions result in higher FA values. Therefore, when there is an overall improvement in the health and environment of a population, a positive trend is typically observed. By contrast, a significant lack of improvement in health and environment tends to result in a negative trend, while no substantial changes lead to a null trend. In addition, secular trends in the size and shape of the cranium and post crania can affect FA measurements and confound results. With regard to the South African population, negative and null trends have been noted. Limited secular studies have been conducted on the South African population, with the majority focusing on the crania of black South Africans, while the post crania have been studied in white South Africans. In a study conducted by Grine et al. (2020), secular trends were studied in the crania of black South Africans born between 1880 and 1959; they observed null secular trends in cranial length but noted a negative trend in cranial breadth in males only. However, Cameron et al. (1990) found a null secular trend for the same features in the same population. As a result, it is not expected that secular trends will have a huge bearing or might slightly inflate the FA results seen in this study for black South Africans. With regard to the white South African population, Myburgh et al. (2017) conducted a study looking at stature using post crania for individuals born between 1946 and 1995, and no secular changes were observed. The crania of white South Africans have not been sufficiently studied; however, extrapolating from the postcranial literature, changes in secular trends of the cranium are not expected. Therefore, FA seen in the white South African population in this study would not be influenced by secular trends. A possible reason for the lack of secular trends in the black and white South African population is that there has been very little improvement or changes in the living conditions in either population from 1880 to 1995. However, additional research is needed to better understand the secular trends in the cranium in both black and white South Africans incorporating more measurements and traits while taking into consideration SES.

## 5 CONCLUSION

The main focus of this study was to address the limited research on craniofacial asymmetry in the South African population and its implication on anthropological studies. The results indicated that high levels of FA were noted among black South Africans, females, and overall black South African females, thus highlighting the role of environmental (such as low SES and health) and genetic (such as population history and heritability) factors in craniofacial asymmetry and FA. These findings contribute to a better understanding of developmental stress and its effect on biological profile estimation in forensic contexts. Accounting for craniofacial asymmetry and FA is crucial in forensic anthropology, as it can significantly influence the accuracy of biological profile estimations, including assessments of sex, ancestry, and facial reconstructions when focusing on shape analyses. Given the historical and socioeconomic disparities in South Africa, further research is essential to explore how these factors influence craniofacial asymmetry and FA in different population groups. Future studies should include larger samples and additional methodologies to improve the understanding of FA in a South African context.

## Supporting information


Data S1.

## Data Availability

The data that support the findings of this study are available upon reasonable request from the corresponding author. The scans are available on the *Bakeng se Afrika* repository. The data from this study are not publicly available due to ethical restrictions.
