# Supplementary material for: Exploring craniofacial fluctuating asymmetry in a South African sample
Source: J Anat. 2025 Apr 23;247(2):314–34. doi: 10.1111/joa.14256 (PMC12265031; doi:10.1111/joa.14256)
Supplement: Supplementary file 1 — Data S1. [file JOA-247-314-s001.docx]

**Table A1** - Descriptive statistics for left and right inter-landmark distances (mm) per sex (F: females, M: males), population affinity (B: black, W: white) and sex/population subgroups (BF: black female, BM: black male, WF: white female, WM: white male). Refer to Table 2 for measurement definitions.

|  | **Sex** | |  | **Population** | |  | **Sex/Population** | | | |  | **Total** |
| --- | --- | --- | --- | --- | --- | --- | --- | --- | --- | --- | --- | --- |
|  | **F (N=57)** | **M (N=58)** |  | **B (N=59)** | **W (N=56)** |  | **BF (N=30)** | **BM (N=29)** | **WF (N=27)** | **WM (N=29)** |  | **(N=115)** |
| **OBH_r** |  |  |  |  |  |  |  |  |  |  |  |  |
| **Mean (SD)** | 36.50 (2.18) | 36.27 (2.41) |  | 36.05 (2.29) | 36.73 (2.27) |  | 36.19 (2.32) | 35.90 (2.29) | 36.84 (2.01) | 36.64 (2.51) |  | 36.38 (2.29) |
| **Min - max** | 31.20 - 41.45 | 29.50 - 41.75 |  | 29.50 - 40.31 | 33.11 - 41.75 |  | 31.20 - 40.31 | 29.50 - 40.17 | 33.68 - 41.45 | 33.11 - 41.75 |  | 29.50 - 41.75 |
| **OBH_l** |  |  |  |  |  |  |  |  |  |  |  |  |
| **Mean (SD)** | 36.09 (2.08) | 36.45 (2.29) |  | 35.70 (2.29) | 36.86 (1.91) |  | 35.42 (2.06) | 35.99 (2.51) | 36.83 (1.86) | 36.89 (1.99) |  | 36.26 (2.19) |
| **Min - max** | 31.48 - 39.99 | 29.97 - 43.41 |  | 29.97 - 43.41 | 32.52 - 42.54 |  | 31.48 - 38.75 | 29.97 - 43.41 | 32.52 - 39.99 | 33.32 - 42.54 |  | 29.97 - 43.41 |
| **NOR_r** |  |  |  |  |  |  |  |  |  |  |  |  |
| **Mean (SD)** | 52.82 (2.50) | 55.28 (2.77) |  | 54.25 (2.42) | 53.86 (3.35) |  | 53.07 (2.38) | 55.47 (1.79) | 52.54 (2.65) | 55.09 (3.51) |  | 54.06 (2.90) |
| **Min - max** | 48.02 - 57.99 | 50.35 - 62.66 |  | 48.02 - 58.84 | 48.73 - 62.66 |  | 48.01 - 57.99 | 52.26 - 58.84 | 48.73 - 57.84 | 50.35 - 62.66 |  | 48.02 - 62.66 |
| **NOR_l** |  |  |  |  |  |  |  |  |  |  |  |  |
| **Mean (SD)** | 53.47 (2.48) | 55.56 (2.73) |  | 54.64 (2.65) | 54.40 (2.98) |  | 53.49 (2.46) | 55.83 (2.32) | 53.44 (2.56) | 55.29 (3.12) |  | 54.52 (2.80) |
| **Min - max** | 46.79 - 59.14 | 49.83 - 61.14 |  | 46.79 - 60.51 | 48.42 - 61.14 |  | 46.79 - 58.14 | 50.79 - 60.51 | 48.42 - 59.14 | 49.83 - 61.14 |  | 46.79 - 61.14 |
| **FMTN_r** |  |  |  |  |  |  |  |  |  |  |  |  |
| **Mean (SD)** | 55.96 (2.21) | 58.28 (3.04) |  | 57.98 (2.83) | 56.24 (2.70) |  | 56.36 (2.12) | 59.66 (2.49) | 55.52 (2.26) | 56.90 (2.94) |  | 57.13 (2.89) |
| **Min - max** | 51.31 - 61.09 | 52.31 - 64.46 |  | 52.11 - 64.46 | 51.31 - 62.70 |  | 52.11 - 61.09 | 55.72 - 64.46 | 51.31 - 59.34 | 52.31 - 62.70 |  | 51.31 - 64.46 |
| **FMTN_l** |  |  |  |  |  |  |  |  |  |  |  |  |
| **Mean (SD)** | 56.33 (2.26) | 58.43 (3.13) |  | 58.29 (2.99) | 56.44 (2.54) |  | 56.83 (2.20) | 59.80(2.97) | 55.78 (2.24) | 57.05 (2.69) |  | 57.39 (2.92) |
| **Min - max** | 49.80 - 60.73 | 51.24 - 65.39 |  | 49.80 - 65.39 | 51.24 - 61.53 |  | 49.80 - 60.40 | 54.94 - 65.39 | 51.92 - 60.73 | 51.24 - 61.53 |  | 49.80 - 65.39 |
| **FMTNS_r** |  |  |  |  |  |  |  |  |  |  |  |  |
| **Mean (SD)** | 74.42 (2.94) | 77.70 (4.09) |  | 75.84 (4.14) | 76.33 (3.68) |  | 74.12 (2.79) | 77.61 (4.59) | 74.76 (3.12) | 77.79 (3.61) |  | 76.08 (3.92) |
| **Min - max** | 69.05 - 81.72 | 64.63 - 85.11 |  | 64.63 - 85.11 | 69.43 - 84.65 |  | 69.05 - 81.72 | 64.63 - 85.11 | 69.43 - 81.54 | 70.41 - 84.65 |  | 64.63 - 85.11 |
| **FMTNS_l** |  |  |  |  |  |  |  |  |  |  |  |  |
| **Mean (SD)** | 74.65 (3.07) | 77.99 (3.82) |  | 76.36 (3.89) | 76.32 (3.82) |  | 74.74 (2.77) | 78.03 (4.20) | 74.55 (3.41) | 77.96 (3.47) |  | 76.34 (3.84) |
| **Min - max** | 67.73 - 81.62 | 68.96 - 86.08 |  | 68.96 - 86.08 | 67.73 - 83.31 |  | 70.24 - 81.62 | 68.96 - 86.08 | 67.73 - 80.93 | 70.52 - 83.31 |  | 67.73 - 86.08 |
| **MAH_r** |  |  |  |  |  |  |  |  |  |  |  |  |
| **Mean (SD)** | 21.23 (9.25) | 22.14 (3.33) |  | 22.01 (3.23) | 21.35 (9.37) |  | 20.72 (2.60) | 23.36 (3.30) | 21.81 (13.27) | 20.92 (2.92) |  | 21.69 (6.91) |
| **Min - max** | 15.93 - 87.34 | 14.81 - 30.59 |  | 15.97 - 30.59 | 14.81 - 87.34 |  | 15.97 - 26.45 | 16.79 - 30.59 | 15.93 - 87.34 | 14.81 - 25.92 |  | 14.81 - 87.34 |
| **MAH_l** |  |  |  |  |  |  |  |  |  |  |  |  |
| **Mean (SD)** | 20.91 (9.17) | 22.19 (3.09) |  | 21.66 (3.17) | 21.44 (9.26) |  | 20.15 (2.50) | 23.23 (3.05) | 21.75 (13.14) | 21.16 (2.80) |  | 21.56 (6.82) |
| **Min - max** | 14.30 - 86.80 | 15.92 - 31.06 |  | 14.30 - 31.06 | 15.92 - 86.80 |  | 14.30 - 24.18 | 18.35 - 31.06 | 16.07 - 86.80 | 15.92 - 26.41 |  | 14.30 - 86.80 |
| **MPL_r** |  |  |  |  |  |  |  |  |  |  |  |  |
| **Mean (SD)** | 30.25 (2.85) | 33.08 (3.45) |  | 31.23 (3.66) | 32.15 (3.20) |  | 29.42 (2.86) | 33.10 (3.48) | 31.18 (2.58) | 33.06 (3.49) |  | 31.678 (3.459) |
| **Min - max** | 20.92 - 35.99 | 25.55 - 40.53 |  | 20.92 - 38.86 | 26.32 - 40.53 |  | 20.92 - 35.69 | 25.55 - 38.86 | 27.25 - 35.99 | 26.32 - 40.53 |  | 20.92 - 40.53 |
| **MPL_l** |  |  |  |  |  |  |  |  |  |  |  |  |
| **Mean (SD)** | 30.21 (3.05) | 33.44 (3.47) |  | 31.43 (3.90) | 32.28 (3.31) |  | 29.41 (3.18) | 33.51 (3.49) | 31.11 (2.68) | 33.37 (3.50) |  | 31.84 (3.63) |
| **Min - max** | 22.44 - 38.98 | 24.75 - 41.96 |  | 22.44 - 40.72 | 27.08 - 41.96 |  | 22.44 - 36.68 | 24.75 - 40.72 | 27.27 - 38.98 | 27.08 - 41.96 |  | 22.44 - 41.96 |
| **MPB_r** |  |  |  |  |  |  |  |  |  |  |  |  |
| **Mean (SD)** | 31.49 (3.42) | 33.61 (5.59) |  | 32.93 (5.31) | 32.17 (4.07) |  | 31.20 (3.24) | 34.72 (6.41) | 31.81 (3.64) | 32.50 (4.48) |  | 32.56 (4.75) |
| **Min - max** | 20.95 - 38.54 | 23.30 - 50.11 |  | 24.88 - 50.11 | 20.95 - 39.59 |  | 25.10 - 37.46 | 24.88 - 50.11 | 20.95 - 38.54 | 23.30 - 39.59 |  | 20.95 - 50.11 |
| **MPB_l** |  |  |  |  |  |  |  |  |  |  |  |  |
| **Mean (SD)** | 31.52 (3.45) | 33.12 (5.03) |  | 32.63 (3.97) | 32.01 (4.79) |  | 32.03 (3.05) | 33.25 (4.71) | 30.96 (3.83) | 32.99 (5.42) |  | 32.33 (4.38) |
| **Min - max** | 23.76 - 38.18 | 24.16 - 45.07 |  | 25.14 - 42.98 | 23.76 - 45.07 |  | 27.56 - 38.18 | 25.14 - 42.98 | 23.76 - 38.14 | 24.16 - 45.07 |  | 23.76 - 45.07 |
| **MSAST_r** |  |  |  |  |  |  |  |  |  |  |  |  |
| **Mean (SD)** | 50.66 (5.31) | 55.34 (6.10) |  | 52.05 (5.47) | 54.04 (6.73) |  | 49.16 (4.49) | 55.05 (4.78) | 52.31 (5.74) | 55.64 (7.26) |  | 53.02 (6.17) |
| **Min - max** | 41.56 - 63.69 | 42.02 - 77.13 |  | 41.74 - 71.86 | 41.56 - 77.13 |  | 41.74 - 56.63 | 47.34 - 71.86 | 41.56 - 63.69 | 42.02 - 77.13 |  | 41.56 - 77.13 |
| **MSAST_l** |  |  |  |  |  |  |  |  |  |  |  |  |
| **Mean (SD)** | 50.02 (4.98) | 55.62 (5.90) |  | 50.94 (5.21) | 54.85 (6.41) |  | 48.16 (3.79) | 53.81 (4.96) | 52.10 (5.37) | 57.42 (6.30) |  | 52.85 (6.12) |
| **Min - max** | 42.95 - 66.16 | 43.41 - 74.47 |  | 42.95 - 64.87 | 44.19 - 74.47 |  | 42.95 - 54.93 | 43.41 - 64.87 | 44.19 - 66.16 | 46.87 - 74.47 |  | 42.95 - 74.47 |
| **OCL_r** |  |  |  |  |  |  |  |  |  |  |  |  |
| **Mean (SD)** | 21.09 (2.40) | 22.48 (2.65) |  | 20.67 (2.18) | 22.97 (2.53) |  | 20.03 (1.89) | 21.33 (2.28) | 22.26 (2.40) | 23.63 (2.51) |  | 21.79 (2.61) |
| **Min - max** | 16.09 - 29.93 | 17.62 - 29.33 |  | 16.09 - 29.33 | 17.62 - 29.93 |  | 16.09 - 24.23 | 17.64 - 29.33 | 18.65 - 29.93 | 17.62 - 27.57 |  | 16.09 - 29.93 |
| **OCL_l** |  |  |  |  |  |  |  |  |  |  |  |  |
| **Mean (SD)** | 21.60 (2.82) | 22.58 (2.85) |  | 20.99 (2.34) | 23.26 (2.92) |  | 20.28 (2.01) | 21.72 (2.46) | 23.06 (2.90) | 23.44 (2.99) |  | 22.09 (2.86) |
| **Min - max** | 16.34 - 30.20 | 17.03 - 30.39 |  | 16.34 - 27.57 | 17.03 - 30.39 |  | 16.34 - 24.40 | 18.86 - 27.57 | 17.81 - 30.20 | 17.03 - 30.39 |  | 16.34 - 30.39 |
| **OPO_r** |  |  |  |  |  |  |  |  |  |  |  |  |
| **Mean (SD)** | 72.97 (3.37) | 76.83 (3.31) |  | 73.62 (3.65) | 76.28 (3.61) |  | 71.56 (3.15) | 75.76 (2.82) | 74.54 (2.93) | 77.90 (3.46) |  | 74.92 (3.85) |
| **Min - max** | 64.78 - 80.66 | 69.88 - 85.32 |  | 64.77 - 80.07 | 70.09 - 85.32 |  | 64.77 - 78.43 | 69.88 - 80.07 | 70.09 - 80.66 | 72.65 - 85.32 |  | 64.77 - 85.32 |
| **OPO_l** |  |  |  |  |  |  |  |  |  |  |  |  |
| **Mean (SD)** | 72.97 (2.96) | 76.53 (3.47) |  | 73.60 (3.37) | 76.00 (3.61) |  | 71.69 (2.65) | 75.56 (2.90) | 74.39 (2.67) | 77.51 (3.76) |  | 74.77 (3.68) |
| **Min - max** | 65.00 - 80.60 | 70.47 - 84.50 |  | 65.00 - 80.64 | 70.01 - 84.50 |  | 64.99 - 77.28 | 70.47 - 80.64 | 70.01 - 80.60 | 72.15 - 84.50 |  | 65.00 - 84.50 |
| **BAPO_r** |  |  |  |  |  |  |  |  |  |  |  |  |
| **Mean (SD)** | 60.11 (3.01) | 63.86 (3.25) |  | 60.76 (3.15) | 63.32 (3.70) |  | 59.11 (2.56) | 62.46 (2.79) | 61.23 (3.12) | 65.26 (3.11) |  | 62.00 (3.64) |
| **Min - max** | 53.60 - 66.65 | 57.78 - 71.51 |  | 53.60 - 67.90 | 54.63 - 71.51 |  | 53.60 - 63.03 | 58.49 - 67.90 | 54.63 - 66.65 | 57.78 - 71.51 |  | 53.60 - 71.51 |
| **BAPO_l** |  |  |  |  |  |  |  |  |  |  |  |  |
| **Mean (SD)** | 60.80 (2.91) | 63.85 (3.18) |  | 61.36 (3.38) | 63.37 (3.13) |  | 59.71 (2.69) | 63.08 (3.20) | 62.02 (2.69) | 64.62 (3.01) |  | 62.34 (3.40) |
| **Min - max** | 53.64 - 68.86 | 57.11 - 70.65 |  | 53.64 - 69.76 | 57.85 - 70.65 |  | 53.64 - 64.62 | 57.11 - 69.76 | 57.85 - 68.86 | 57.93 - 70.65 |  | 53.64 - 70.65 |
| **NMS_r** |  |  |  |  |  |  |  |  |  |  |  |  |
| **Mean (SD)** | 121.33 (4.90) | 127.73 (6.11) |  | 124.20 (6.40) | 124.93 (6.41) |  | 120.54 (4.38) | 127.98 (6.00) | 122.20 (5.36) | 127.48 (6.33) |  | 124.55 (6.39) |
| **Min - max** | 110.81 - 131.24 | 115.69 - 144.13 |  | 113.90 - 139.50 | 110.81 - 144.13 |  | 113.90 - 129.24 | 115.87 - 139.50 | 110.81 - 131.24 | 115.69 - 144.13 |  | 110.81 - 144.13 |
| **NMS_l** |  |  |  |  |  |  |  |  |  |  |  |  |
| **Mean (SD)** | 120.89 (4.92) | 128.24 (5.65) |  | 123.93 (6.36) | 125.30 (6.51) |  | 120.09 (4.57) | 127.90 (5.49) | 121.78 (5.24) | 128.58 (5.89) |  | 124.30 (6.44) |
| **Min - max** | 111.21 - 133.12 | 111.52 - 143.13 |  | 111.21 - 136.08 | 112.64 - 143.13 |  | 111.21 - 130.50 | 111.52 - 136.08 | 112.64 - 133.12 | 118.67 - 143.13 |  | 111.21 - 143.13 |
| **NAL_r** |  |  |  |  |  |  |  |  |  |  |  |  |
| **Mean (SD)** | 43.45 (3.48) | 46.26 (3.95) |  | 43.44 (3.74) | 46.36 (3.67) |  | 42.05 (2.97) | 44.88 (3.94) | 44.99 (3.40) | 47.64 (3.50) |  | 44.86 (3.97) |
| **Min - max** | 37.31 - 51.21 | 36.07 - 54.40 |  | 36.07 - 52.25 | 38.13 - 54.40 |  | 37.31 - 48.90 | 36.07 - 52.25 | 38.13 - 51.21 | 40.81 - 54.40 |  | 36.069 - 54.401 |
| **NAL_l** |  |  |  |  |  |  |  |  |  |  |  |  |
| **Mean (SD)** | 43.18 (3.32) | 45.34 (4.05) |  | 42.79 (3.59) | 45.83 (3.50) |  | 41.72 (2.61) | 43.90 (4.15) | 44.81 (3.31) | 46.78 (3.45) |  | 44.27 (3.85) |
| **Min - max** | 36.71 - 52.06 | 33.87 - 52.14 |  | 33.87 - 52.14 | 37.48 - 52.06 |  | 36.71 - 47.59 | 33.87 - 52.14 | 37.48 - 52.06 | 37.74 - 51.35 |  | 33.87 - 52.14 |
| **NAAL_r** |  |  |  |  |  |  |  |  |  |  |  |  |
| **Mean (SD)** | 16.84 (1.89) | 17.73 (1.88) |  | 17.349 (1.956) | 17.22 (1.92) |  | 16.10 (1.70) | 17.71 (2.16) | 16.66 (2.11) | 17.74 (1.59) |  | 17.29 (1.93) |
| **Min - max** | 13.54 - 21.26 | 12.83 - 22.30 |  | 12.83 - 22.30 | 13.54 - 21.26 |  | 13.74 - 19.98 | 12.83 - 22.30 | 13.54 - 21.26 | 14.79 - 20.35 |  | 12.83 - 22.30 |
| **NAAL_l** |  |  |  |  |  |  |  |  |  |  |  |  |
| **Mean (SD)** | 16.62 (1.92) | 18.00 (1.85) |  | 17.47 (1.90) | 17.15 (2.11) |  | 16.96 (1.59) | 18.00 (2.07) | 16.23 (2.20) | 18.00 (1.64) |  | 17.31 (2.00) |
| **Min - max** | 11.77 - 20.13 | 12.93 - 21.48 |  | 12.93 - 21.48 | 11.77 - 21.07 |  | 13.48 - 20.13 | 12.93 - 21.48 | 11.77 - 19.83 | 15.15 - 21.07 |  | 11.77 - 21.48 |
